# Extracellular vesicles as prospective biological indicators for midgestational placental complications in the mouse

**DOI:** 10.3389/fcell.2025.1636335

**Published:** 2025-07-24

**Authors:** Elaine Lee, Parinaz Kazemi, Shiva Shafiei, Sarah Yull, Mansuba Rana, Nadim Tawil, Laura Montermini, Janusz Rak, Daniel Dufort

**Affiliations:** ^1^Division of Experimental Medicine, McGill University, Montreal, QC, Canada; ^2^Child Health and Human Development Program, Research Institute of the McGill University Health Centre, Montreal, QC, Canada; ^3^Department of Obstetrics and Gynecology, McGill University, Montreal, QC, Canada; ^4^Department of Pediatrics, McGill University, Montreal, QC, Canada

**Keywords:** pregnancy, placenta, proteomics, miRNAs, Nodal, exosomes, EVs

## Abstract

**Background:**

Placental dysfunction is often associated with reproductive complications such as preeclampsia, intrauterine growth restriction (IUGR), and preterm birth. Currently, the early diagnosis and intervention of these pathologies remain challenging due to the invasive nature of placental tissue sampling. Liquid biopsies of extracellular vesicles (EVs) released from the placenta have emerged as a prospective minimally invasive diagnostic strategy that could provide insight into the maternal-fetal interface because of the active role EVs play in mediating placental development and function. However, the lack of information on EVs directly from placenta at disease onset has questioned the representativeness of placental EVs as pathological indicators. To address these concerns, this study assessed the accuracy with which tissue-derived D10.5 placental EVs could identify phenotypes exhibited by a reproductively challenged Nodal conditional knockout mouse model at mid-gestation.

**Method:**

Implantation sites from female mice with a uterine-specific knockdown of the Nodal gene were examined from D8.5 to D14.5 utilizing histological analysis, Western blotting, and RT-qPCR to characterize their mid-gestational phenotypes. Placental EVs were then isolated from D10.5 placenta using enzymatic digestion, differential centrifugation, filtration, and size-exclusion chromatography. The final EV fractions were concentrated and validated with size analysis, canonical protein markers, and morphology assessment. Differential expression analysis across the EV samples was performed using proteomics and miRNA-Seq. Functional enrichment analysis of dysregulated EV factors was then completed using several gene ontology databases along with a literature review to determine whether placental EVs could indicate the reproductive abnormalities presented by the mutant mice.

**Results:**

Uterine-specific deletion of Nodal resulted in IUGR and fetal loss in mutant dams. Decidualization and placentation defects were observed, including thinner decidual and placental tissues, impaired angiogenesis, and an altered junctional zone within the maternal-fetal interface. Bioinformatics analysis of EV cargo identified 31 differentially expressed proteins and 10 miRNAs specifically linked to placental development, oxidative stress, angiogenesis, and immunomodulation. Notably, 15 of these proteins and six of these miRNAs have been previously associated with pregnancy complications, further supporting the prospects of placental EVs as biomarkers for various placental diseases.

**Conclusion:**

These findings suggest that placental EVs can reflect compromised placental function and could serve as pathological indicators for the early detection of pregnancy complications. Their potential diagnostic utility could improve maternal and neonatal health outcomes by enabling earlier intervention and monitoring of high-risk pregnancies.

## 1 Introduction

By mediating resource exchange, immunotolerance, and various other physiological adaptations across the maternal-fetal interface, the placenta distinguishes itself as an organ essential for fetal development and pregnancy maintenance (Burton and Fowden, 2015; Ding et al., 2022; Gude et al., 2004; Smith et al., 2021). Compromised placental formation and function have been recurrently linked to several pregnancy complications such as intrauterine growth restriction (IUGR), preeclampsia, and preterm birth, which serve as leading causes for the high maternal and neonatal mortality rates observed worldwide (Hemberge et al., 2020; Norwitz, 2006). While direct analysis of the placental tissue via chorionic villus sampling (CVS) has been proposed as an effective method to detect reproductive pathologies, the risks associated with the invasiveness of this technique, such as bleeding, infection, membrane rupture, amniotic fluid leakage, fetal limb defects and pregnancy loss, have largely deterred the prenatal employment of such procedures for diagnostic purposes (Jones and Montero, 2022; Salomon et al., 2019). Because of this, there is a significant unmet need for a minimally invasive approach that can analyze the placenta early in pregnancy to better diagnose and monitor emerging complications, maximizing prevention and treatment opportunities for expectant mothers.

Extracellular vesicles (EVs) are cell-derived nanoparticles that are generated through the inward invagination of early endosomes or by the outward blebbing of the plasma membrane (van Niel et al., 2018). Functionally, they participate in intercellular communication and regulate numerous biological processes through the exchange of proteins, small RNAs, and lipids (Hessvik and Llorente, 2018). In the context of mammalian reproduction, EVs have demonstrated active roles in precipitating pathological phenotypes frequently associated with placental complications. One study found that procoagulant EVs from activated endothelial cells could reduce embryo size, diminish placental diameter, and promote proteinuria in mice at mid-pregnancy by upregulating the trophoblastic expression of inflammasome markers, while another found that EVs from hypoxic trophoblasts could impair the angiogenic capacity of endothelial cells by suppressing the chondroitin polymerizing factor pathway (Kohli et al., 2016; Sha et al., 2023). Analysis of EVs from term preeclamptic placentas also revealed the differential expression of cargo components like decreased endothelial nitric oxide synthase levels which was associated with increased vasoconstriction and hypertension, both characteristic features of preeclampsia (Motta-Mejia et al., 2017). The discovery of various mechanisms and molecular mediators by which EVs propagate the pathophysiology of placenta-related complications has encouraged the exploration of their clinical applicability. In particular, the recent discovery of EVs that retain the biological signature of their tissue of origin in the systemic circulation has highlighted their potential as liquid biopsy disease biomarkers that can indicate dysfunction at the maternal-fetal interface for the diagnosis of numerous conditions in a minimally-invasive manner that circumvents the risks associated with currently available techniques (Zeng et al., 2022).

Although several groups have attempted to isolate placental EVs from the plasma of patients with pregnancy complications using the placental alkaline phosphatase (PLAP) or syncytin-1 tissue markers for this exact purpose, the inability to compare these EVs with those directly from the developing placenta at the time of disease onset has challenged their validity as representative tissue factors capable of capturing the pathological environment responsible for the complication in question (Chaemsaithong et al., 2023; Levine et al., 2020). We aimed to address these uncertainties and explore the feasibility of utilizing EVs for the early diagnosis of reproductive pathologies by first determining if tissue-derived placental EVs could accurately capture mid-gestational phenotypes exhibited by an established mouse model for pregnancy complications. In this study, we characterized the mid-pregnancy phenotypes exhibited by dams with a uterine-specific knockout of the Nodal gene, which have previously displayed various adverse reproductive outcomes like implantation failure early in gestation to an increased susceptibility to inflammation and preterm birth during late pregnancy (Ayash et al., 2020; Yull et al., 2023). Because Nodal and its signaling pathway have demonstrated active roles in regulating trophoblast differentiation, invasion, and proliferation, we rationalized that the morphogen would be an ideal gene target for modeling complications related to abnormal placentation (Ma et al., 2001; Law et al., 2014; Munir et al., 2004). Tissue-derived placental EVs from these mice were then collected at mid-pregnancy and analyzed to identify protein and microRNA (miRNA) cargo components that could account for the observed pathological phenotypes. By assessing the potential of EVs as biological indicators of placental dysfunction, we hope our findings can ultimately advance the development of novel diagnostic approaches to improve reproductive outcomes for patients with high-risk pregnancy complications.

## 2 Materials and methods

### 2.1 Generation of Nodal mutant mice

All experimental and animal handling protocols were approved by the Animal Care Committee at the Research Institute of the McGill University Health Centre (AUP #: MUHC-5261) and adhered to the regulations stipulated by the Canadian Council on Animal Care. To produce a mouse model that exhibited pregnancy abnormalities, adult female dams with a uterine-specific knockout of the *Nodal* gene were generated using the loxP-Cre recombinase system as previously described (Park et al., 2012). Mice with loxP sites flanking exons 2 and 3 of the *Nodal* gene (Nodal^loxP/loxP^) on a mixed background were generously donated by E. J. Robertson (University of Oxford) while progesterone receptor (PR)-Cre mice (Pgr^Cre/+^) on a C57BL6/129 background were provided by F. J. DeMayo and J. P. Lydon (Baylor College of Medicine) (Lu and Robertson, 2004; Soyal et al., 2005). Nodal^loxP/loxP^ mice were crossed with Pgr^Cre/+^ mice and appropriate breeding pairs were formed from subsequent offspring generations to generate the control females (Nodal^loxP/loxP^, Pgr^+/+^ henceforth referred to as Nodal^loxP/loxP^) and the homozygous *Nodal* mutant females (Nodal^loxP/loxP^, Pgr^Cre/+^ henceforth referred to as Nodal^∆/∆^) used in this study.

### 2.2 Mating and manipulation of transgenic mice

Nodal^loxP/loxP^ and Nodal^∆/∆^ females aged 8–24 weeks were mated overnight with wild-type CD1 males (Charles River Laboratories). The presence of a vaginal plug the following morning was designated as day 0.5 of pregnancy (D0.5). On day 8.5 (D8.5), day 10.5 (D10.5), day 12.5 (D12.5), and day 14.5 (D14.5) of pregnancy the females were euthanized, and uterine horns were collected in 1 × RPMI 1640 media (350-000 CL, WISENT Inc.) on ice.

### 2.3 Whole-mount analysis of embryos and implantation sites

Photographs of whole-mount uteri from the Nodal^loxP/loxP^ and Nodal^∆/∆^ females were taken at D8.5, D10.5, D12.5, and D14.5, where the area of individual implantation sites was measured using the ImageJ software. Embryos were dissected from the whole-mount uteri at D10.5, D12.5, and D14.5; and their morphology was assessed using the Leica M20F4A microscope. Embryos dissected on D14.5 were then dried and weighed.

### 2.4 Tissue processing, paraffin embedding, sectioning, and H&E staining

Dissected uteri were collected in PBS and fixed in 10% neutral buffered formalin at 4°C for at least 48 h. After fixation, tissues were processed using an automated tissue processing machine (Leica ASP300S) and embedded in paraffin wax. Paraffin blocks were solidified on a cold plate for 1 h and then stored at −20°C until sectioning. Sections of 7 μm thickness were prepared using a Leica RM2145 microtome where they were then mounted and dried on Fisher Superfrost Plus slides. Slides were then either used for Hematoxylin and Eosin (H&E) staining or immunofluorescence.

### 2.5 Immunofluorescence

Slides were deparaffinized in xylene twice for 10 min each and rehydrated through graded ethanol concentrations. Antigen retrieval was performed in Tris-EDTA (pH 9) at 95°C for 25 min. Slides were rinsed in permeabilization buffer twice (TBS, 0.2% BSA, 0.025%–0.25% Triton X-100) for 5 min, then incubated overnight at 4°C with primary antibodies for Placental lactogen I (PL1) (sc-34713, Santa Cruz, stock concentration 0.2 mg/mL, diluted 1:50 in 1% BSA/TBS), CD31 (ab182981, Abcam, stock concentration 0.519 mg/mL, diluted 1:1,500 in 1% BSA/TBS), or TPBPA (ab104401, Abcam, stock concentration 0.9 mg/mL, diluted 1:200 in 1% BSA/TBS). After three 5 min interval washes (TBS, 0.05% Tween), slides were incubated with the appropriate secondary antibody, Alexa Fluor 488 (A11055, Invitrogen, diluted 1:300) or Alexa Fluor 594 (R37119, Invitrogen, diluted 1:300), and then counterstained with DAPI (diluted 1:500) for 2 h at room temperature. Slides were subsequently washed three times for 5 min (TBS, 0.05% Tween) and mounted with Richard-Allan Scientific™ Mounting Medium (Thermo Fisher Scientific).

### 2.6 Isolation of placental extracellular vesicles

The decidua and placenta were collected from D10.5 uterine horns, quartered, and digested in 1 × RPMI 1640 media with 20 μg/mL of Liberase™ TM (5401119001, Roche) and 30 μg/mL of DNase I (DN25, Sigma-Aldrich) such that every 0.2 g of tissue was exposed to 2 mL of digestion media (Crescitelli et al., 2021). Samples were incubated for 30 min at 37 ℃ , after which the media was pooled through a 0.70 μm cell strainer and centrifuged sequentially at 500 × *g* and 2,000 × *g*, each for 15 min at 4 ℃ to remove tissue fragments and cell debris (Crescitelli et al., 2021). The supernatant was filtered through a 0.45 μm filter and concentrated to 500 μL using an Amicon Ultra-15 Centrifugal Filter (100 kDa, UFC910024, MilliporeSigma). EVs were isolated using a qEVoriginal size-exclusion chromatography (SEC) column (ICO-70, Izon Sciences Limited), eluted with 0.1- μ m-filtered PBS, and collected in five fractions of 400 μL each. SEC eluates of isolated EVs were then concentrated to 50–70 μL using an Amicon Ultra-0.5 Centrifugal Filter (100 kDa, UFC510024, MilliporeSigma) and stored at −80 ℃ until further analysis was performed.

### 2.7 Nanoparticle tracking analysis (NTA)

To determine the size distribution and concentration of the particles present in the isolated samples, NTA was performed using the NanoSight NS300 instrument (Malvern Panalytical). The samples were injected into the instrument with a 1 mL syringe at a speed of 25 μL/s after being diluted 400- to 2,000-fold using 0.1- μ m-filtered PBS to achieve an optimal particle distribution field of view of 20–80 particles per frame. Five 30-s videos were acquired at a detection threshold of 5, a camera level of 14, a laser wavelength of 532 nm, and a temperature of 37 ℃ . The NanoSight Software NTA 3.4 (version 3.4.4) was used for analysis of instrument’s measurements.

### 2.8 Transmission electron microscopy

To visualize the collected EVs, 5 μL of the SEC isolated EV sample was placed on top of a charged 200-mesh carbon-coated Cu grid for 5 min. The grid was washed three times with distilled water and then negatively stained with 2% uranyl acetate for 45 s to 1 min. Excess solution present on the grid was blotted off using Whatman filter paper, and the grid was air-dried for 1 h prior to imaging. The imaging was performed using the FEI Tecnai 12 BioTwin 120 kV TEM (Thermo Fisher Scientific Inc.) with the AMT XR80C CCD Camera System (AMT Imaging) at the Facility for Electron Microscopy Research of McGill University.

### 2.9 Protein extraction and concentration quantification

Protein was extracted from placental tissues isolated from each implantation site at D8.5 and D10.5 for both the Nodal^loxP/loxP^ and Nodal^∆/∆^ females. Tissues were homogenized in 1 mL of 1 × RIPA buffer (9806, Cell Signaling Technology, Inc.) that contained 1 × Protease Inhibitor Cocktail (04693159001, Roche) and 2 mM of PMSF (P7626-1G, Sigma-Aldrich) and incubated on a plate rocker for 15 min at 4 ℃ . After centrifuging for 10 min at 10,000 × *g* at 4 ℃ , the middle supernatant layer that contained protein was collected and its concentration was measured using the Pierce™ BCA Protein Assay Kit (23225, Thermo Fisher Scientific Inc.). To extract protein from EVs, the isolated EV samples were treated with the 1 × RIPA buffer in a 1:1 ratio. Protein concentration was then determined using the Micro BCA™ Protein Assay Kit. Absorbance for concentration quantification was measured at 562 nm.

### 2.10 Western blotting

The concentrations of the samples were normalized with filtered PBS, then mixed 1:1 with 2 × Laemmli buffer containing β -mercaptoethanol and heated at 95 ℃ for 5 min for protein denaturation. The proteins were resolved on a 12% SDS-PAGE gel and transferred to a 0.2 μm PVDF membrane (1620177, Bio-Rad Laboratories, Inc.). The membranes were blocked with 5% non-fat skim milk in 1 × TBS-T and subsequently incubated overnight with the primary antibodies of interest. CD31 expression was quantified using anti-CD31 (ab182981, Abcam, 1:2,000) and EV markers were detected with anti-CD63 (143901, BioLegend, 1:500), anti-TSG101 (934301, BioLegend, 1:500), anti-CD9 (124802, BioLegend, 1:500), and anti-HSC70 (sc-7298, Santa Cruz Biotechnology, Inc., 1:500) respectively. Intracellular contamination was assessed with anti-Calnexin (sc-32249; Santa Cruz Biotechnology, Inc., 1:500). After washing, membranes were incubated with the appropriate secondary antibody, rinsed, and treated with the Clarity™ Western ECL Substrate (1705061, Bio-Rad Laboratories, Inc.) for 5 min before being imaged using the Amersham™ Imager 600 (GE Healthcare Technologies, Inc.). Bands were quantified using the ImageJ software.

### 2.11 Liquid chromatography-mass spectrometry (LC-MS) proteomics

LC-MS proteomics analysis was performed by the Proteomics and Molecular Analysis Platform at the Research Institute of the McGill University Health Centre (RI-MUHC). The protein concentrations of the EV samples (N = 4 Nodal^loxP/loxP^; N = 4 Nodal^∆/∆^) were normalized to 67 μg per sample. Proteins were resolved in a stacking gel band, reduced with DTT, alkylated with iodoacetic acid, and digested with trypsin. Extracted peptides were re-solubilized in 0.1% aqueous formic acid and loaded onto an Acclaim™ PepMap™ precolumn (75 μM ID × 2 cm C18 3 μM beads, Thermo Fisher Scientific Inc.) and then onto an Acclaim™ PepMap™ EASY-Spray™ analytical column (75 μM × 15 cm with 2 μM C18 beads, Thermo Fisher Scientific Inc.). The peptides were then separated using a Dionex UltiMate 3000 UHPLC (Thermo Fisher Scientific Inc.) at 250 nL/min with a gradient of 2%–35% organic (0.1% formic acid in acetonitrile) over 3 h. Peptides were analyzed using a Thermo Scientific™ Orbitrap Fusion™ mass spectrometer (Thermo Fisher Scientific Inc.) operating at 120,000 resolution (FWHM in MS1) with HCD sequencing (15,000 resolution) at top speed for all peptides with a charge of 2+ or greater. The raw data was converted into a *.mgf format (Mascot generic format) so it can be searched in the Mascot 2.6.2 search engine (Matrix Science) to delineate mouse protein sequences (UniProt, 2023).

### 2.12 Tissue RNA extraction and RT^2^ quantitative PCR profiler array

Decidual and placental tissues from four implantation sites were isolated from each female on D10.5 then pooled and stored at −80°C until used. Samples were homogenized, and total RNA was extracted using Trizol (15596018, Invitrogen) and the RNeasy Mini Kit (74104, Qiagen). The RT2 First Strand Kit (330401, Qiagen) was used for cDNA synthesis. Reverse transcription quantitative PCR (RT-qPCR) was performed using the RT^2^ SYBR Green qPCR Mastermix (330500, Qiagen) and the Mouse Pre-Eclampsia (PAMM-163Z) profiler array (330231, Qiagen) following the manufacturer’s protocol. The 96-well array contained 84 genes of interest, five housekeeping genes, one internal control for genomic DNA contamination, three reverse transcription controls for assessment of RNA quality, and three positive controls for general determination of PCR performance. Samples were run on the Roche LightCycler 480 thermocycler and analyzed using Qiagen GeneGlobe software. Relative expression of the genes of interest was calculated by the ΔΔCt method, where the average fold change in Nodal^Δ/Δ^ gene expression was relative to the Nodal^loxP/loxP^ mice and normalized to the five housekeeping genes (*Actb*, *B2m*, *Gapdh*, *Gusb* and *Hsp90ab1*).

### 2.13 EV small RNA extraction

To extract total RNA, the EV samples (N = 3 Nodal^loxP/loxP^; N = 3 Nodal^∆/∆^) were first treated with 20 μg/μL of proteinase K for 30 min at 37 ℃ to purify the samples of non-EV protein contamination (Ibrahim et al., 2024). The samples were then treated with 20 μg/μL of RNAse A (FEREN0531, Fisher Scientific International) for 2 min at room temperature to eliminate any non-EV RNA contamination (Ibrahim et al., 2024). The QIAZol Lysis Reagent from the Qiagen miRNeasy Micro Kit (217084, QIAGEN N.V.) was added to the samples in a 5:1 ratio and vortexed for 5 s before being kept at room temperature for 5 min. Chloroform was then added to the samples in a 1:5 volume ratio, vortexed for 15 s, and left to incubate for 3 min at room temperature. The samples were centrifuged for 15 min at 12,000 × *g* at 4 ℃ , and the resulting aqueous phase was removed and mixed with 1.5 × 100% ethanol. The samples were transferred to the MinElute™ columns from the Qiagen miRNeasy Micro Kit and the instructions outlined in kit were then followed to complete the RNA extraction.

### 2.14 miRNA library generation and sequencing

Quantification of the total RNA concentration, generation of the small RNA libraries, and the miRNA sequencing were performed by the Centre d’expertise et de services Génome Québec. The total RNA concentration was quantified using a NanoDrop Spectrophotometer ND-1000 (NanoDrop Technologies, Inc.). Libraries were generated from 1,000 ng of total RNA using the NEBNext Multiplex Small RNA Library Prep Kit for Illumina (New England Biolabs), as per the manufacturer’s recommendations. cDNA construct purification was performed using SparQ beads (QIAGEN N.V.). Libraries were quantified using the KAPA Library Quantification Kits - Complete kit (Universal) (Kapa Biosystems) and the average size fragment was determined using a Fragment Analyzer instrument (Agilent Technologies, Inc.). The libraries were normalized and pooled. The pool was diluted to 650 pM using RSB Tween20 and a phiX library was used as a control and mixed with the libraries at a 5% level. The pool was loaded on a P1 NextSeq 2000 lane as per the manufacturer’s instructions (Illumina, Inc.) and the run was performed for 2 × 100 cycles (paired-end mode). Program BCL Convert 4.2.4 was then used to demultiplex samples and generate FASTQ reads. The raw FASTQ files were initially processed with fastp to remove adapter sequences and ensure quality control. The resulting trimmed reads were subsequently input into the exceRpt pipeline for small RNA sequence identification and transcript quantification.

### 2.15 Bioinformatics and statistical analysis

For the proteomics data, the Mascot 2.6.2 search results were loaded onto the Scaffold Q+/Scaffold 5 software (version 5.2.2, Proteome Sciences) for quantitative analysis. Proteins with peptide reads missing in >50% of the replicates per group were removed. The resulting protein list was checked against the National Center for Biotechnology Information (NCBI) Gene database (National Center for Biotechnology Information, 2004) and the TissueEnrich webtool (Jain and Tuteja, 2021), which both utilized transcriptome data from the Mouse ENCODE Project to determine the tissue specificity of each protein. The statistical significance of a tissue’s enrichment score was calculated using the hypergeometric test with Benjamini-Hochberg correction. Differential expression analysis was performed on Scaffold by calculating fold changes and applying the Student’s t-test. Proteins were differentially expressed between the Nodal^loxP/loxP^ and Nodal^∆/∆^ EVs if they displayed a fold change > 1.5 and a p < 0.05. Enriched gene ontology and phenotype terms were identified through over-representation analysis using the PANTHER (version 18.0) and MOET (version 2.0) online databases respectively, where the statistical significance of each term was determined with the Fischer’s exact test or the hypergeometric test (Mi et al., 2019; Thomas et al., 2022; Vedi et al., 2022).

For the miRNA-Seq results, small RNAs not classified as miRNAs and those with transcript reads missing in >1/3 of the replicates per group were filtered out. Differential expression analysis was performed with the DESeq2 R package using the raw count data generated by the exceRpt pipeline. The log_2_ fold change difference was calculated, and statistical significance was determined using the Wald test with Benjamini-Hochberg correction (adjusted p < 0.05, log_2_ fold change > 1.5). miRNA-gene interactions were annotated using the multiMiR R package, which incorporated the miRTarBase database for validated target information. Associated target genes were subjected to over-representation analysis to elucidate enriched gene ontology and phenotype terms using the clusterProfiler R package and the MOET online database. Figures were generated using the ggplot2 and pheatmap packages on R version 4.3.1.

A systematic literature review was performed for proteins and miRNAs differentially expressed between the two groups to determine if they had any previously reported roles in placentation or related processes.

For other experiments, statistical analyses were performed in GraphPad Prism (v9.4.1) using the two-tailed unpaired Student’s t-test. Data was presented as mean ± SD or mean ± SEM, with significance set at p < 0.05.

## 3 Results

### 3.1 Dams with a uterine-specific knockout of Nodal exhibit intrauterine growth restriction and fetal loss during mid-pregnancy

**FIGURE 1:**
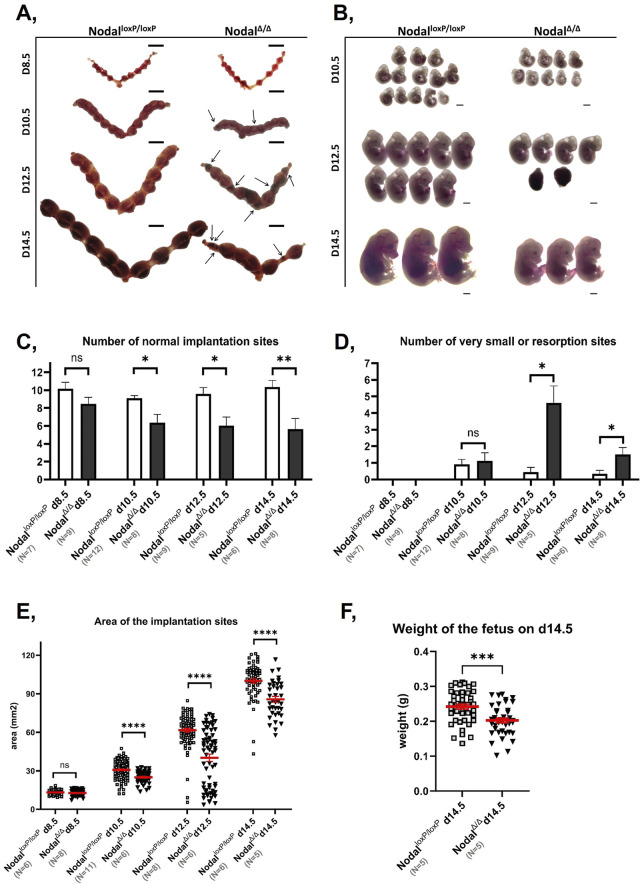
Nodal^∆/∆^ dams are affected by IUGR and fetal loss during mid-pregnancy: **(A)** Representative photographs of whole mount uteri from Nodal^loxP/loxP^ (left) and Nodal^∆/∆^ mice (right) at D8.5, D10.5, D12.5 and D14.5 of gestation (from top to bottom). Arrows indicate resorbed implantation sites and scale bar represents 10 mm. **(B)** Representative photographs of embryos extracted from Nodal^loxP/loxP^ (left) and Nodal^∆/∆^ dams (right) at D10.5, D12.5 and D14.5 of gestation (from top to bottom). Scale bar represents 1 mm. **(C, D)** Bar graphs comparing the **(C)** number of morphologically normal implantation sites and **(D)** number of very small or resorbed sites between the Nodal^loxP/loxP^ and Nodal^∆/∆^ mice at D8.5, D10.5, D12.5, and D14.5 of gestation. Values are shown as the mean number of implantation sites ± SEM. **(E)** Dot plot comparing area measurements of implantation sites from the Nodal^loxP/loxP^ and Nodal^∆/∆^ mice at D8.5, D10.5, D12.5, and D14.5 of gestation. **(F)** Dot plot comparing fetal weight measurements of embryos from the Nodal^loxP/loxP^ and Nodal^∆/∆^ dams at D14.5 of gestation. *p < 0.05, **p < 0.01, ***p < 0.001 and ****p < 0.0001 indicate significant differences between the Nodal^loxP/loxP^ and Nodal^∆/∆^ mice.

The generation of a mouse model with a uterine-specific deletion of the *Nodal* gene was achieved by utilizing the *Pgr*-Cre and Nodal^loxP/loxP^ strains as previously described (Park et al., 2012). In previous studies, Nodal deficiency in the maternal reproductive tract was found to limit the fecundity of affected females as indicated by the reduced litter sizes produced by these dams (Yull et al., 2023; Park et al., 2012). Although the subfertility observed in 50% of the knockout females was previously attributed to implantation failure that occurred prior to the establishment of pregnancy, the diminished reproductive capacity of the remaining pregnant Nodal^∆/∆^ dams was postulated to be a result of mid-gestational fetal loss (Yull et al., 2023). To confirm this, Nodal^loxP/loxP^ controls and Nodal^Δ/Δ^ knockout females were mated with wild-type CD1 males, and their reproductive tracts were examined at D8.5, D10.5, D12.5, and D14.5 post-coitum. At D8.5, there were no significant differences in the whole-mount appearance (Figure 1A), or in the number (Figures 1C, D) or size (Figure 1E) of the implantation sites between the two groups. However, the number of normal implantation sites was significantly lower in the Nodal^Δ/Δ^ females by D10.5 (Figures 1A, C) with an increase in resorbed sites by D12.5 (Figures 1D, E). Further assessment also revealed that embryos from Nodal^Δ/Δ^ mice were morphologically smaller throughout mid-pregnancy and exhibited significantly lower weights by D14.5 compared to embryos from the Nodal^loxP/loxP^ dams (Figures 1B, F). Overall, the subfertility of those Nodal^Δ/Δ^ females that were successfully able to implant was found to be due to fetal growth restriction and fetal loss that manifested during mid-pregnancy.

### 3.2 Nodal deficient dams exhibit abnormalities in the development and function of the maternal-fetal interface

To better understand the underlying pathophysiology for the observed fetal phenotypes, the maternal-fetal interface of the Nodal^Δ/Δ^ mice was analyzed due to its extensive role in supporting fetal development and pregnancy maintenance (Burton and Fowden, 2015; Gude et al., 2004). When looking at H&E-stained cross sections of D10.5, D12.5, and D14.5 implantation sites from the Nodal^Δ/Δ^ females, the thickness of the maternal decidua basalis (MD) was found to be significantly reduced across all timepoints, which indicated decidualization abnormalities in these mice compared to the Nodal^loxP/loxP^ dams (Supplementary Figures S1A, B). Similarly, the fetal placental tissues (FP) of the Nodal^Δ/Δ^ dams were also found to be significantly thinner at D12.5 and D.14.5, which suggested that placentation was compromised in these females as well (Supplementary Figures S1A, C).

**FIGURE 2:**
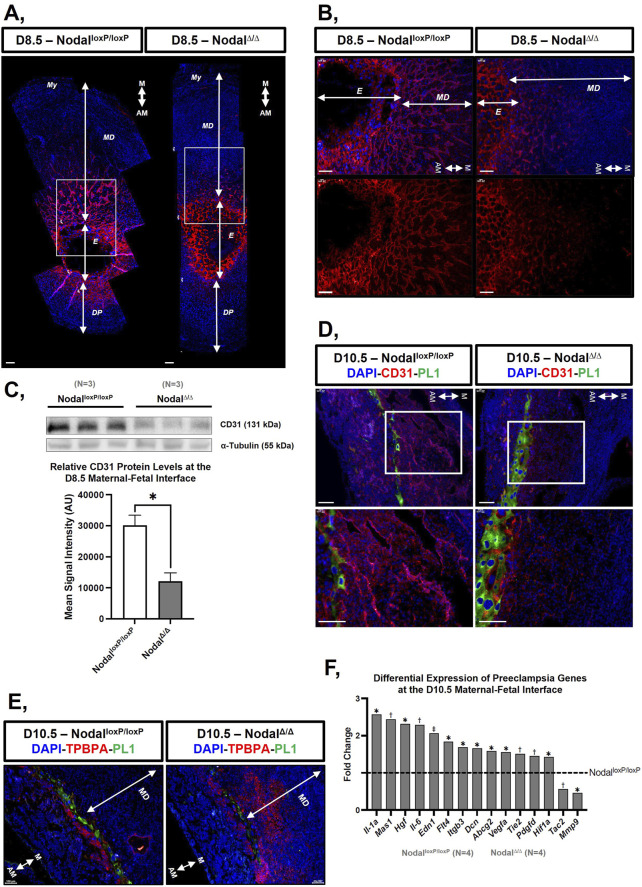
The maternal-fetal interface of the Nodal^∆/∆^ dams exhibits vascular development deficiencies, expansion of trophoblast giant cell layer and an altered gene expression profile at mid-pregnancy: **(A, B)** Immunofluorescence staining of sectioned implantation sites containing the maternal uterus, maternal decidua, and fetal placenta from the Nodal^loxP/loxP^ and Nodal^∆/∆^ mice at D8.5 with endothelial cell marker CD31 (red) and DAPI (blue). **(B)** Is higher magnification of the area marked by white rectangles in **(A)**. **(C)** Western blot and corresponding bar graph quantifying CD31 protein expression in the implantation sites from the Nodal^loxP/loxP^ and Nodal^∆/∆^ mice at D8.5. Values on the bar graph display the mean signal intensity of CD31 normalized against α-tubulin ± SD and *p < 0.05 indicates a significant difference. **(D)** Immunofluorescence staining of sectioned implantation sites from the Nodal^loxP/loxP^ and Nodal^∆/∆^ mice at D10.5 with trophoblast giant cell marker PL1 (green), CD31 (red) and DAPI (blue). Bottom images are a higher magnification of the area marked by white rectangles in the top images. **(E)** Immunofluorescence staining of sectioned implantation sites from the Nodal^loxP/loxP^ and Nodal^∆/∆^ mice at D10.5 with PL1 (green), spongiotrophoblast marker TPBPA (red) and DAPI (blue). **(F)** Bar graph illustrating the differential expression of various genes from the 84-gene Mouse Pre-Eclampsia RT^2^ profiler array between the Nodal^loxP/loxP^ and Nodal^∆/∆^ mice at the D10.5 maternal-fetal interface where *p < 0.05, ^†^p < 0.01, and ^‡^p < 0.001 indicate significant differences. Scale bars represent 100 μm. Different layers within the implantation site are indicated as My, myometrium; MD, maternal decidua basalis; E, embryonic originated cells; DP, decidua parietalis. The orientation of the tissue is indicated by M, mesometrial; AM, anti-mesometrial.

Morphological differences in the Nodal^Δ/Δ^ tissues suggested impairments in the development of the maternal-fetal interface, prompting further investigation into the maternal response to embryo implantation and placentation. One major change that normally occurs during the formation of the maternal-fetal interface is an upregulation in angiogenesis within the decidual and placental tissues (Hemberge et al., 2020; Simmons et al., 2014; Watson and Cross, 2005). Although this typically occurs simultaneously with uterine decidualization around D5.5, immunofluorescence analysis with endothelial cell marker CD31 revealed disrupted vascularization in the Nodal^Δ/Δ^ maternal-fetal interface at mid-pregnancy. Compared to the Nodal^loxP/loxP^ controls, CD31 staining was markedly reduced showing restricted distribution from the periphery of the embryo to the mesometrial pole of the implantation site from D8.5 to D10.5 (Figures 2A, B). Western blot analysis confirmed the significantly lower CD31 protein levels in the D8.5 implantation sites of the Nodal^Δ/Δ^ females (Figure 2C). Because proper angiogenesis is essential for establishing a vascular network by which resource exchange can occur across the maternal-fetal interface, these findings not only highlighted an additional pathological phenotype, but they also elucidated a potential mechanism by which Nodal deletion in the maternal reproductive tract could compromise fetal growth and pregnancy progression in affected dams.

In addition to increased vascular remodeling, the formation of the maternal-fetal interface involves extensive cell differentiation and controlled trophoblast invasion (Hemberge et al., 2020; Simmons et al., 2014; Watson and Cross, 2005). In particular, 1 cell type that is essential for integrating the placenta with the decidua to establish a physiological connection between the mother and fetus during pregnancy are the parietal trophoblast giant cells (pTGCs) (Hemberge et al., 2020; Watson and Cross, 2005). Probing D10.5 implantation sites with placental lactogen (PL1), pTGC marker, showed inconsistencies in the development of the trophoblast giant cell layers between the Nodal^loxP/loxP^ and Nodal^∆/∆^ females. The latter had either an increased number of PL1 positive cells (Figure 2D) or a deficient number of PL1 positive cells with an increased number of cells expressing the trophoblast specific protein alpha (TPBPA) marker for spongiotrophoblasts (Figure 2E), which are also pTGC progenitors (Simmons et al., 2007). Overall, the dysregulated differentiation of pTGCs suggested that trophoblast invasion was disturbed in these dams as well, which was further illustrated by the extensive expansion of TPBPA positive spongiotrophoblasts into the maternal decidua from the junctional zones of the Nodal^∆/∆^ mice (Figure 2E).

To further determine how the maternal-fetal interface was compromised in the Nodal^Δ/Δ^ dams at mid-pregnancy, an RT^2^ Profiler Array was used to assess the expression of genes associated with biological processes pertinent to pregnancy and placentation within the D10.5 implantation sites. Out of 84 genes, 13 were significantly upregulated (*Abcg2*, *Dcn*, *Edn1*, *Flt4*, *Hgf*, *Hif1a*, *Il-1a*, *Il-6*, *Igtb3*, *Mas1*, *Pdgfd*, *Tie2*, and *Vegfa*) while the expression of two genes (*Mmp9* and *Tac2*) was decreased in the Nodal^Δ/Δ^ mice compared to Nodal^loxP/loxP^ control females (Figure 2F). Among the differentially expressed genes, many were found to be involved in angiogenesis and trophoblast invasion, which reinforced the vascular and trophoblast abnormalities seen in the Nodal^Δ/Δ^ females at mid-pregnancy (Gerhardt et al., 2003; Hu et al., 2024; Lu et al., 2022; Zhang et al., 2020). Furthermore, some of the dysregulated genes were discovered to have other established roles in processes normally tightly regulated by the placenta, such as blood vessel contractility, hormone signaling, oxidative stress, hypoxia and inflammation, which confirmed that the functionality of the maternal-fetal interface was severely compromised in the Nodal^Δ/Δ^ mice as well (Kiowski et al., 1991; Li et al., 2019; Vukovic et al., 2001; Werman et al., 2004) (Table 1).

**TABLE 1:** Differentially expressed preeclamptic genes at the D10.5 maternal-fetal interface of the Nodal^Δ/Δ^ females.

Gene	Fold change	P-value	Description
*Il1a*	5.74	0.0016	LOPE, pro-inflammatory cytokine, angiogenesis
*Mas1*	2.44	0.0051	LOPE, vasodilation, angiogenesis
*Hgf*	2.32	0.0126	EOPE, growth factor, trophoblast invasion
*Il6*	6.56	0.0179	Pro-inflammatory cytokine, angiogenesis
*Edn1*	2.06	0.0006	Vasoconstriction, angiogenesis
*Flt4*	1.84	0.0445	Angiogenesis
*Itgb3*	1.69	0.0319	LOPE, trophoblast invasion, angiogenesis
*Dcn*	1.66	0.0284	EOPE, pregnancy maintenance, decidualization, angiogenesis
*Abcg2*	1.58	0.0174	EOPE, LOPE, hemostasis
*Vegfa*	1.56	0.0127	Angiogenesis
*Tek/Tie2*	1.51	0.0026	Angiogenesis, trophoblast growth and migration
*Pdgfd*	1.45	0.0025	LOPE, growth factor, angiogenesis
*Hif1a*	1.42	0.0305	Hypoxia, oxidative stress, pregnancy maintenance, angiogenesis
*Tac2*	0.56	0.0034	Growth factor, pregnancy maintenance, hypoxia
*Mmp9*	0.46	0.0113	Tissue remodeling, trophoblast invasion, decidualization, angiogenesis, oxidative stress

Description of 15 differentially expressed genes between the Nodal^Δ/Δ^ (N = 4) and Nodal^loxP/loxP^ (N = 4) females with established pathophysiological roles in preeclampsia (LOPE, late onset preeclampsia; EOPE, early onset preeclampsia). 13 genes were significantly upregulated and 2 were downregulated in the Nodal^Δ/Δ^ dams. Data was derived from an 84-gene RT^2^ profiler array of factors related to preeclampsia pathogenesis. P-value <0.05 was considered statistically significant based on the two-tailed unpaired Student’s t-test.

### 3.3 Nanoparticles extracted from maternal-fetal interface exhibit quantitative and qualitative features characteristic of EVs with a placenta-specific tissue signature

To assess whether the midgestational phenotypes displayed by the Nodal^Δ/Δ^ mice could accurately be reflected by EVs, the cargo of EVs derived from the maternal-fetal interface of D10.5 pregnant dams was analyzed. Although some of the placental abnormalities exhibited by the Nodal^∆/∆^ mice were present earlier in gestation as well, we postulated that D10.5 would be a better analysis timepoint because of the more robust development of the maternal-fetal interface at this stage, which would magnify and enhance the detection of the observed phenotypes.

**FIGURE 3:**
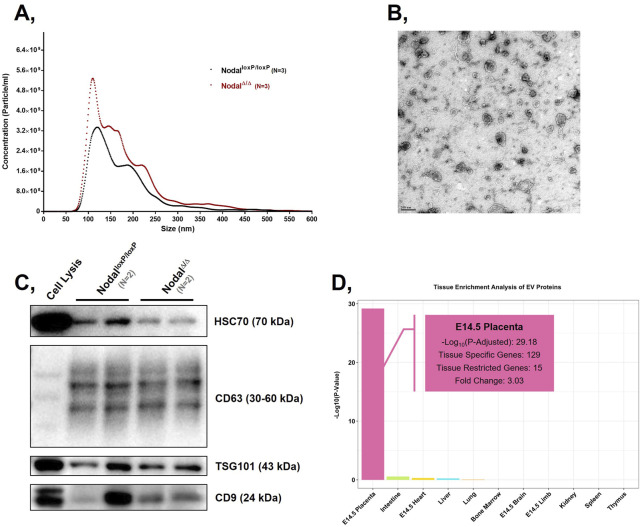
Collected particles exhibit size, morphological features, and protein markers characteristic of placental EVs: **(A)** Graphical representation of the concentration and size distribution of the particles present in the Nodal^loxP/loxP^ and Nodal^∆/∆^ EV samples isolated from enzymatically digested D10.5 placentas. **(B)** TEM image of Nodal^loxP/loxP^ EVs from enzymatically digested D10.5 placentas. Scale bar represents 200 nm in the image. **(C)** Western blots displaying the expression of EV markers Hsc70, Cd63, Tsg101, and Cd9 in Nodal^loxP/loxP^ and Nodal^∆/∆^ EV samples isolated from D10.5 placentas. D10.5 placental cell lysate (“Cell Lysis”) was used as a positive control. Approximately 25 μg of total protein was loaded across all samples. **(D)** Bar graph comparing the enrichment score of proteins from the Nodal^loxP/loxP^ (N = 4) and Nodal^∆/∆^ (N = 4) EV samples across 11 different mouse tissues. The enrichment score is expressed on a −Log_10_ (p-value) scale.

First, to confirm the isolation of EVs, NTA, TEM, and immunoblotting were performed to verify EV parameters as per the guidelines of the 2023 Minimal Information for Studies of Extracellular Vesicles (MISEV) (Welsh et al., 2024). Based on the NTA results, the particles in the Nodal^loxP/loxP^ and Nodal^Δ/Δ^ samples had a mean diameter of 173.6 ± 11 and 171.4 ± 16.2 nm respectively (Figure 3A), which were both within the typical EV size range (van Niel et al., 2018; Welsh et al., 2024). Furthermore, the concentration of particles in the Nodal^loxP/loxP^ and Nodal^Δ/Δ^ samples were approximately 3.5e+11 particles/mL and 5.3e+11 particles/mL respectively, which confirmed the comparable efficacy of the isolation protocol for both groups. TEM analysis allowed us to visualize membranous spherical structures in the samples (Figure 3B), which resembled previously published images of EVs (Rikkert et al., 2019). Along with the confirmed expression of common EV markers CD63, CD9, TSG101, and HSC70 in the samples via immunoblotting (Figure 3C), these findings further validated the successful isolation of EVs from the tissues of the maternal-fetal interface by the established protocols (Kowal et al., 2016). It is important to note that while these orthogonal measures confirmed the samples possessed EVs, they also revealed the possibility of co-isolated intracellular material. This was illustrated by the presence of non-EV aggregates in the TEM images as well as the faint Western blot detection of calnexin in the samples (Supplementary Figure S2), which may reflect residual endoplasmic reticulum contamination. Although this challenged the absolute purity of the collected samples, the positive EV indications cumulatively provided by the NTA, TEM, and Western blot data strongly supported that the samples primarily consisted of EVs.

To confirm that the collected EVs reflected their source tissue of origin, they were analyzed for markers distinctive to the placenta. Because placenta-specific miRNAs, such as those from the chromosome 19 miRNA cluster (C19MC), have only been found in primates thus far, the tissue source of the extracted EVs were characterized by protein markers (Bentwich et al., 2005). When mapping the isolated proteins to a database consortium that provided transcriptomic profiles on various murine tissues at D14.5, the proteomic landscape of the EVs was most highly enriched for placental proteins by a fold change of 3 and with a statistically significant p-value of 10^–29^ (Figure 3D). From a total of 1,446 proteins, 129 were previously found to be elevated in the placenta while 15 had exhibited expression patterns specifically restricted to this reproductive tissue. Upon further analysis, the proteins exclusively expressed in the placenta were also found to be characteristic markers of various placental cell types (Table 2). For example, PRL3D1 was reported as a common parietal trophoblast giant cell marker while CTSJ was associated with trophoblasts found within the labyrinthine layer (Nakajima et al., 2000; Simmons et al., 2008). Overall, through the identification of numerous placenta-specific proteins via tissue enrichment analysis, the samples were confirmed to contain EVs that were derived from a placental source.

**TABLE 2:** Placenta-specific proteins identified from the Nodal^loxP/loxP^ and Nodal^Δ/Δ^ EVs.

Protein	Placental cell type
PRL2B1	S-TGC, SpT (Simmons et al., 2014; Simmons et al., 2008)
PRL2C2, PRL2C3, PRL2C5	C-TGC, P-TGC, SpA-TGC (Simmons et al., 2014; Simmons et al., 2007; Simmons et al., 2008)
PRL3B1	C-TGC, P-TGC, S-TGC, SpT (Simmons et al., 2014; Simmons et al., 2007; Simmons et al., 2008)
PRL3D1	P-TGC (Simmons et al., 2014; Simmons et al., 2007; Simmons et al., 2008)
PRL4A1	P-TGC, SpA-TGC (Simmons et al., 2014; Simmons et al., 2008)
PRL7A1	P-TGC, SpT (Simmons et al., 2008)
PRL7D1	C-TGC, P-TGC, S-TGC, SpA-TGC, SpT, GlyT (Simmons et al., 2008)
GZMD, GZME, GZMF, GZMG	GlyT, uNK, decidua (Sferruzzi-Perri et al., 2009; Kaur et al., 2022)
PSG22	TGC (Williams et al., 2015)
CTSJ	Labyrinth trophoblast cells (Nakajima et al., 2000)

15 EV proteins collected from the Nodal^loxP/loxP^ and Nodal^Δ/Δ^ mice were exclusively expressed in the placenta. The placental cell types where each of these proteins are most highly expressed are specified. TGC, trophoblast giant cell; S-TGC, sinusoidal trophoblast giant cell; P-TGC, parietal trophoblast giant cell; C-TGC, canal trophoblast giant cells; SpA-TGC, spiral artery trophoblast giant cell; SpT, spongiotrophoblast; GlyT, glycogen trophoblast; uNK, uterine natural killer cell.

### 3.4 Placental EVs from Nodal^Δ/Δ^ females at mid-pregnancy differentially express proteins and miRNAs that are involved in biological processes related to placentation and placental dysfunction

**FIGURE 4:**
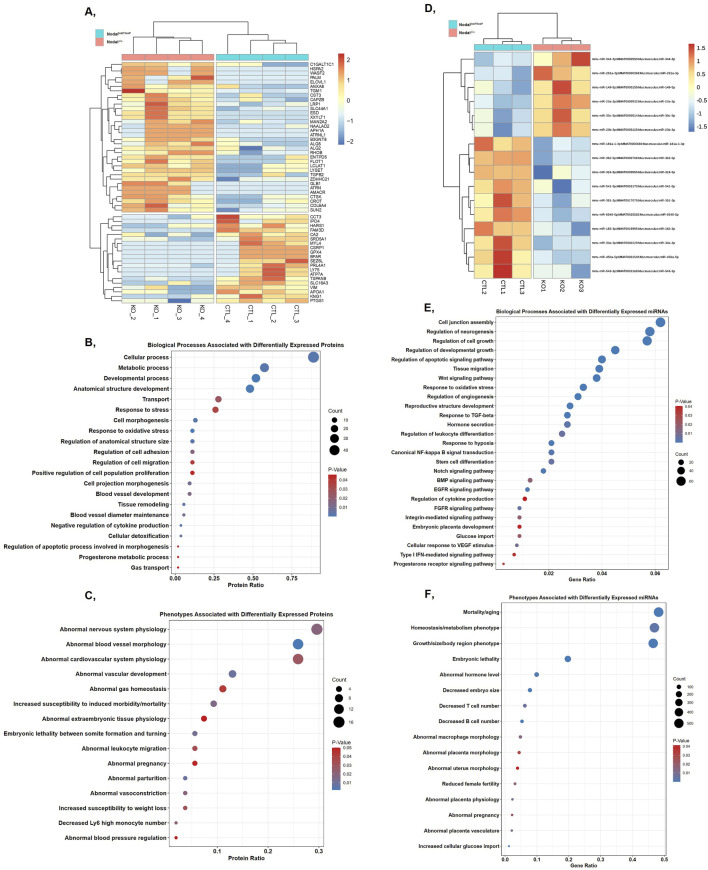
Comparing the expression, biological functions, and associated phenotypes of EV cargo factors differentially expressed between the Nodal^loxP/loxP^ and Nodal^∆/∆^ mice at D10.5: **(A)** Heatmap displaying the expression and hierarchical clustering of proteins differentially expressed between the Nodal^loxP/loxP^ and Nodal^∆/∆^ EV samples with a fold-change > 1.5 and a p < 0.05. Proteins were clustered using the Euclidean distance metric and complete linkage agglomeration method. Colour key denotes z-score normalized peptide counts where blue to red indicates low to high protein expression levels respectively. **(B)** Placentation-related GO biological processes and **(C)** placental phenotypes associated with the proteins differentially expressed between the Nodal^loxP/loxP^ and Nodal^∆/∆^ EVs. **(D)** Heatmap displaying the expression and hierarchical clustering of miRNAs differentially expressed between the Nodal^loxP/loxP^ and Nodal^∆/∆^ EV samples with a log_2_ (fold-change) > 1.5 and a p < 0.05. Clustering was based on correlation distance and the complete linkage agglomeration method. Colour key denotes VST transformed transcript counts where blue to red indicates low to high expression levels respectively. **(E)** Placentation-related GO biological processes and **(F)** placental phenotypes associated with the target genes of miRNAs differentially expressed between the Nodal^loxP/loxP^ and Nodal^∆/∆^ EVs. For the graphs, the bubble size indicates the number of proteins or miRNA target genes affiliated with each term while the colour indicates its specific enrichment score (p-value), although p < 0.05 for all listed terms. The top three terms based on protein or target gene count are also included for all graphs to illustrate the relative enrichment of the terms of interest compared to others associated with differentially expressed EV cargo components.

When analyzing the protein cargo of the isolated EVs, 54 proteins were found to be significantly differentially expressed between the Nodal^loxP/loxP^ and Nodal^Δ/Δ^ EVs (Figure 4A). Of these proteins, 34 were upregulated in the Nodal^Δ/Δ^ EVs while 20 were downregulated. GO analysis revealed that many of the EV proteins differentially expressed between the two groups were associated with biological processes relevant for the morphological development of the placenta as well as its role as a modulator of the tissue’s vascular and immunological microenvironment. Specifically, EV proteins differentially expressed between the two strains were enriched for GO terms pertaining to “anatomical structure development” (VIM, APOA1, PRL4A1), “transport” (CA2, SLC44A1, SLC16A3), “regulation of anatomical structure size” (KNG1, PTGS1, ATP7A), “regulation of cell migration” (RHOB, VIM, SUN2), “blood vessel development” (LRP1, WASF2, RHOB), and “negative regulation of cytokine production” (APOA1, TGFB2) (Figure 4B). Additionally, when mapping these proteins against the Mammalian Phenotype Ontology database, some interesting phenotypic terms that appeared included “abnormal vascular development” (RHOB, WASF2, APH1A), “abnormal extraembryonic tissue physiology” (PRL4A1, ATP7A, WASF2), “abnormal leukocyte migration” (ANXA8, GLB1, VIM), “embryonic lethality between somite formation and embryo turning” (ALG5, CCT3, GPX4), and “abnormal pregnancy” (GPX4, PTGS1, SRD5A1) (Figure 4C).

miRNA-Seq analysis of the EV transcriptome revealed 16 miRNAs that were significantly differentially expressed between the Nodal^loxP/loxP^ and Nodal^Δ/Δ^ EVs (Figure 4D). Among the differentially expressed miRNAs, six were upregulated in the Nodal^Δ/Δ^ EVs while 10 were downregulated. These miRNAs targeted a total of 1,094 genes where 497 were specifically targeted by the upregulated EV miRNAs, 511 by the downregulated miRNAs, and 86 by both. GO analysis of the target genes revealed that differentially expressed miRNAs between the two groups were associated with similar biological processes to those implicated by the proteomics results, such as “regulation of developmental growth” (miR-149-5p, miR-291a-3p), “tissue migration” (miR-541-5p, miR-30c-5p), “regulation of angiogenesis” (miR-324-5p, miR-362-5p), “regulation of cytokine production” (miR-23a-3p, miR-541-5p), and “embryonic placenta development” (miR-149-5p, miR-30c-5p) (Figure 4E). Mapping the target genes of the differentially expressed miRNAs against the Mammalian Phenotype Ontology database also revealed an enrichment in many interesting terms, such as “embryonic lethality” (miR-362-5p, miR-541-5p), “decreased embryo size” (miR-362-5p, miR-149-5p), “abnormal placenta morphology” (miR-362-5p, miR-149-5p), “abnormal placenta physiology” (miR-149-5p, miR-30c-5p), “abnormal placenta vasculature” (miR-362-5p, miR-30c-5p), and “reduced female fertility” (miR-23b-3p, miR-362-5p) (Figure 4F). Similar to the proteomics data, these findings further highlighted the capacity with which placental EV miRNAs could reflect underlying dysfunction in placental development.

**FIGURE 5:**
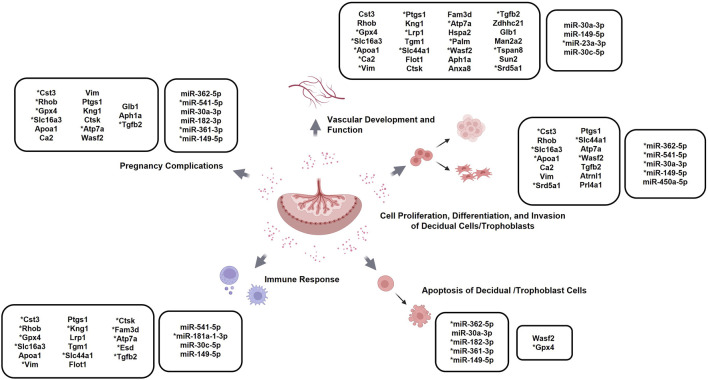
Specific EV cargo factors with established roles in placentation-related processes and associated complications: Schematic highlighting proteins and miRNAs differentially expressed between the placental EVs from the Nodal^loxP/loxP^ and Nodal^∆/∆^ mice at D10.5 with previously reported roles in pregnancy complications and biological processes related to placentation, such as angiogenesis, immune responses, and trophoblast differentiation. *denotes placental EV factors whose reported expression-specific effects coincides with those observed in the Nodal^∆/∆^ dams. Created in BioRender.com.

To further validate the bioinformatic findings and the overall feasibility of utilizing EVs as potential biomarkers for placental defects, we cross-referenced the differentially expressed EV cargo factors with existing literature. Among the differentially expressed cargo components, 31 proteins and 10 miRNAs were identified with previously reported roles in pregnancy complications, placentation, or other related biological processes (Figure 5) (Bahrami et al., 2021; Hao et al., 2015; Wang et al., 2020; Zhang and Zhao, 2021). Some of these processes included those involved in trophoblast invasion and differentiation, decidualization, angiogenesis, and the modulation of tissue immune responses (Supplementary Table S1). Because these factors were functionally implicated in processes also impaired in the Nodal^Δ/Δ^ females, they reinforced the potential utility of placental EVs as indicators of midgestational placental dysfunction.

## 4 Discussion

Because of their demonstrated roles in pregnancy and associated complications, the analysis of EVs released from the maternal-fetal interface has been actively explored as a novel diagnostic strategy for various placental disorders (Kohli et al., 2016; Markmeyer et al., 2021). However, EV factors that have been presented as prospective disease biomarkers thus far have also been questioned for their accuracy due to being obtained from term placentas or trophoblast cell lines, which are limited in their ability to faithfully recapitulate the tissue environment at disease onset (Motta-Mejia et al., 2017; Awoyemi et al., 2024). This study aimed to address these concerns, as well as the feasibility of the proposed diagnostic approach, by assessing whether EVs collected from the pathologically implicated placenta at mid-gestation could correctly reflect the phenotypes displayed by a reproductively challenged mouse model at the same corresponding timepoint.

When exploring the Nodal^∆/∆^ females as disease models for placental complications, we found that many of the reproductive phenotypes exhibited by these dams throughout mid-pregnancy were generally consistent with what was observed in mice that were genetically disrupted for the Nodal signaling pathway or other members of the TGF- β superfamily. For example, in alternative mouse models, the dysregulated expression of *Alk4* or *Cripto* were found to induce fetal loss and IUGR as well (Peng et al., 2015; Shafiei and Dufort, 2021). Previous studies also found that genetically silencing *Smad2/3* could compromise the expression of decidualization markers; excess activin A could increase trophoblast apoptosis; and depleting *Nodal* could impair angiogenesis and promote the expansion of the trophoblast giant cell and spongiotrophoblast layers within the placenta (Ma et al., 2001; Quail et al., 2012; Yu et al., 2012; Zhao et al., 2012). The parallels between these findings with the changes seen in the Nodal^∆/∆^ mice supported the legitimacy of the delineated phenotypes.

Interestingly, an analysis of the D10.5 placental EVs from the Nodal^Δ/Δ^ females highlighted numerous cargo factors whose dysregulated expression could have been indicative of the described midgestational phenotypes because of their reported pathological effects on placental development and function. For example, since the inhibition of SLC16A3 was previously found to decelerate the *in vitro* proliferation and differentiation of progenitor endometrial stromal cells, the downregulated levels of this protein in the Nodal^∆/∆^ EVs could have been representative of the decidualization defects seen in the mutant females (Zuo et al., 2015). Another case in point that demonstrated this was the increased expression of miR-23a-3p in the Nodal^∆/∆^ EVs, which was believed to be indicative of the impaired placental vascularization observed in the mutant dams because of the miRNA’s role as a negative regulator of angiogenesis (Zhou et al., 2024). Further supplementing this, we discovered that the differential expression of certain cargo factors could also be reflective of pathological phenotypes that were presented by the Nodal^Δ/Δ^ mice in preceding studies as well. One EV factor that exemplified this was the CTSK protein as its upregulation was associated with M2 macrophage polarization and the increased secretion of cytokines like TNF- α , IL-6, and IL-1 α , which was concordant with the immunocompromised and highly proinflammatory placental environment previously observed in the Nodal^Δ/Δ^ dams at mid-pregnancy (Hao et al., 2015; Wu et al., 2022; Yull and Dufort, 2025). Overall, the consistency between the dysregulated phenotypes implicated by the EV cargo with those observed in the Nodal^Δ/Δ^ females validated the competency and accuracy with which placental EVs could identify midgestational pathological changes at the maternal-fetal interface.

While not prevalent, it is important to note that the study identified some cargo factors whose dysregulated expression contradicted the pathological phenotypes exhibited by the Nodal^Δ/Δ^ mice based on their previously established functional roles. For example, vimentin, which is a mesenchymal marker commonly associated with enhanced trophoblast invasion, was downregulated in the Nodal^Δ/Δ^ dams, which was inconsistent with the increased expansion of the pTGCs and spongiotrophoblasts seen in the junctional zones of these females at D10.5 (Xiong et al., 2021). Although these discrepancies could challenge the reliability of EVs as indicators for the observed phenotypes, we propose that the differential expression of these factors could also be interpreted as a compensatory response enacted to counteract the pathophysiological changes at the maternal-fetal interface. Such homeostatic mechanisms have been reported in placental tissues before as exemplified by one study that discovered that the increased antioxidant activity displayed in some preeclamptic placentas was actually a mitochondrial adaptation implemented to neutralize the high levels of reactive oxygen species also present in the placental tissues of impacted patients (Holland et al., 2018). With this alternative explanation, even the differential expression of these controversial EV cargo components could have indirectly been indicative of the pathological phenotypes exhibited by the Nodal^Δ/Δ^ dams, which reasserted the ability of the EVs to accurately discern placental dysfunction.

One caveat of the study was that our use of a bioinformatics-exclusive approach impeded us from ascertaining the specific pathological effects of the implicated EV cargo factors within the Nodal^∆/∆^ dams. Although we attempted to cross-check the function of each dysregulated cargo factor with a literature review, the use of various research models, tissues, and experimental conditions in the reference studies meant that the proposed roles of these EV factors within the Nodal^Δ/Δ^ mice were made with a certain degree of assumption and uncertainty (Fitzgerald et al., 2023; Huang et al., 2017; Shaw et al., 2022). Another experimental concern of the study was regarding the clinical applicability of the findings. In this study, the Nodal^∆/∆^ dams were used as models for high-risk pregnancy complications because the placental abnormalities exhibited by these mice were frequently implicated in the pathophysiology of diseases like IUGR and preeclampsia (Bujorescu et al., 2023; Dunk et al., 2019; Proctor et al., 2009; Goulopoulou and Davidge, 2015; Jackson et al., 1995; Kennedy et al., 2020; Opichka et al., 2021). As a result, we had projected that the correct identification of the Nodal^∆/∆^ phenotypes with the placental EVs also indicated their diagnostic potential as disease biomarkers, which was a major extrapolation without further validation with patient samples. Because of these limitations, future studies will have to include functional assays, like cell proliferation assays with trophoblasts, to confirm the biological effects of the implicated EV factors on the Nodal^Δ/Δ^ dams as well as an analysis of plasma-derived placental EVs from human patients with highly sensitive and quantifiable methods like ELISA or RT-qPCR to assess the clinical translatability of our work.

It is worth mentioning that the study’s experimental design also demonstrated some unique strengths. For example, the use of an animal model ensured the analyzed EVs were directly derived from the pathologically implicated placenta at the time of phenotype onset. Unlike previous studies, this approach allowed us to circumvent the concerns regarding the representativeness of the EV source and properly assess the accuracy with which placental EVs could reflect disturbances at the maternal-fetal interface (Motta-Mejia et al., 2017; Awoyemi et al., 2024). Because of this, our findings with the Nodal^Δ/Δ^ mice provided a well-supported foundation and incentive for further exploring the analysis of placental EVs as a novel diagnostic strategy.

## 5 Conclusion

Overall, our study not only characterized a novel research model for midgestational placental complications but highlighted the strength of placental EVs as biological indicators for adverse reproductive outcomes. Parallels between the placental phenotypes bioinformatically implicated by the collected EVs with those observed in the Nodal^Δ/Δ^ dams demonstrated how EVs could correctly reflect the morphological and physiological abnormalities displayed at the maternal-fetal interface during mid-pregnancy. Utilizing the Nodal^Δ/Δ^ mice further validated the accuracy of these demonstrated capabilities as the analyzed EVs were directly obtained from the impacted placenta around the time of phenotype onset. Future studies should aim to confirm the biological relevance and clinical translatability of the identified EV factors as biomarkers of placental dysfunction. However, the presented findings ultimately reaffirmed the potential of harnessing EVs as a novel diagnostic strategy to gain insight into the placenta for the detection of various high-risk pregnancy complications that compromise maternal-fetal health prospects worldwide.

## Data Availability

The miRNA sequencing data generated in this study have been deposited in the Gene Expression Omnibus (GEO) under accession number GSE301046. The mass spectrometry proteomics data have been deposited to the ProteomeXchange Consortium via the PRIDE partner repository with the dataset identifier PXD065500 and DOI 10.6019/PXD065500.
