# Supplementary material for: Extracellular vesicles as prospective biological indicators for midgestational placental complications in the mouse
Source: Front Cell Dev Biol. 2025 Jul 24;13:1636335. doi: 10.3389/fcell.2025.1636335 (PMC12329223; doi:10.3389/fcell.2025.1636335)
Supplement: Supplementary file 1 [file DataSheet1.docx]

**SUPPLEMENTARY MATERIALS**

**SUPPLEMENTAL FIGURE 1**


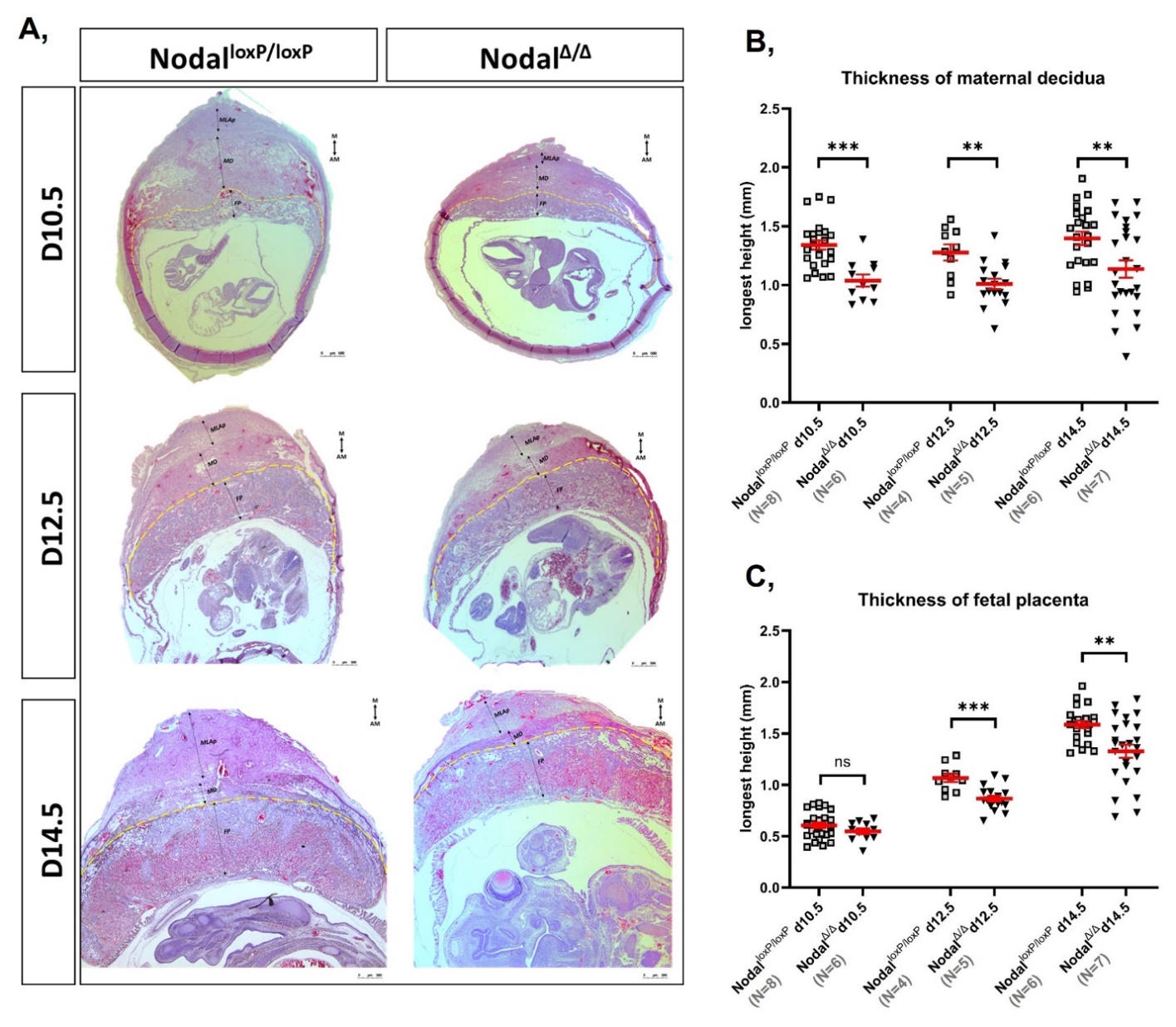


**SUPPLEMENTAL FIGURE 1.** Maternal decidua and fetal compartments of the placenta are underdeveloped in Nodal^Δ/Δ^ females at mid-pregnancy: (A) H&E staining of sectioned implantation sites from the Nodal^loxP/loxP^ (left) and Nodal^∆/∆^ (right) females at D10.5, D12.5, and D14.5 of gestation (from top to bottom). Different layers within the implantation site are indicated as MLAp = mesometrial lymphoid aggregate of pregnancy, MD = maternal decidua basalis, and FP = fetal placenta. The orientation of the tissue is indicated by M = mesometrial and AM = anti-mesometrial. Scale bars represent 500 $\mu$m. (B-C) Dot plots showing the quantitative characterization of the thickness of the (B) maternal decidua and (C) fetal placenta from the Nodal^loxP/loxP^ and Nodal^∆/∆^ females at D10.5, D12.5, and D14.5 of gestation. **p<0.01 and ***p<0.001 indicate significant differences between the Nodal^loxP/loxP^ and Nodal^∆/∆^ females.

**SUPPLEMENTAL FIGURE 2**

**SUPPLEMENTAL FIGURE 2.** Western blot detection of the endoplasmic reticulum marker Calnexin in D10.5 placental cell lysate (positive control) and a representative placental EV sample from Nodal^loxP/loxP^ mice. Calnexin was strongly detected in the cell lysate and faintly detected in the EV preparation.

**Supplemental Table 1. Functional roles of placental EV proteins and miRNAs of interest:** Description of select EV cargo factors (31 proteins and 10 miRNAs) differentially expressed between the Nodal^loxP/loxP^ and Nodal^Δ/Δ^ mice with previously characterized roles in pregnancy or related processes.

| EV Cargo Factor | | P-Value | Reported Roles in Pregnancy & Associated Processes |
| --- | --- | --- | --- |
| Proteins | **CST3** | p = 4.8E-02  ($\downarrow$ CTL, $\uparrow$ KO) | - Decreased expression of decidual markers Prl and Igfbp1 in hormonally treated endometrial stromal cells (1) - Blocking Cst3 increased the proliferation, migration, and invasion of trophoblasts (2) - Blocking also reduced the embryo resorption rate and increased the weight of fetuses and placentas in mice with recurrent spontaneous abortion (2) - Cst3 overexpression induced VEGF-mediated angiogenesis (3) - Found to enhance inflammation by increasing NF$\kappa$B p65 activation and the production of NO and TNF$\alpha$ in macrophages (4) |
|  | **RHOB** | p = 4.1E-02  ($\downarrow$ CTL, $\uparrow$ KO) | - Genetic knockdown in human endometrial stromal cells inhibited physiological changes necessary for decidualization (i.e., actin cytoskeletal rearrangement, cell morphological transformation, and expression of decidual marker IGFBP1) (5) - Implicated in impairing placental microvilli outgrowth to induce preeclamptic symptoms by promoting shedding of syncytiotrophoblast microparticles (6) - Suppression decreased endothelial cell sprouting via increased apoptosis, reduced tube formation, and impaired endothelial cell migration by interfering with the organization of actin into stress fibers (7,8) - Could be a marker for hypoxia as it exhibited increased expression and activity in human pulmonary artery endothelial and smooth muscle cells under hypoxic conditions (9) - Increased production of proinflammatory cytokines IL-1$\beta$, IL-6, and TNF-$\alpha$ by increasing NF$\kappa$B activity in macrophages (10) - Inhibition promoted macrophage M2 polarization (11) |
|  | **GPX4** | p = 2.5E-02  ($\uparrow$ CTL, $\downarrow$ KO) | - Inhibition promoted ferroptosis in primary human trophoblasts and during mouse pregnancy (12) - Degradation promoted ferroptosis of HUVECs and downregulated expression of proangiogenic Vegfa and Ang-1 (13) - Supplementation reversed miscarriage phenotype in mouse model (13) - Downregulated in placental tissue samples from preeclamptic patients (14,15) - Protects neutrophils and macrophages from lipid peroxidation, ferroptosis, or necrosis (16,17) - Deficiency in Tregs increased superoxide and proinflammatory cytokines (i.e., IL-1$\beta$, CCL19, CCL6, CXCL2, CCL5, CSF1) (18) |
|  | **SLC16A3** | p = 1.7E-02  ($\uparrow$ CTL, $\downarrow$ KO) | - Expressed in the microvillous membrane of syncytiotrophoblasts as a marker of syncytiotrophoblast layer II cells (19,20) - Major lactate transporter within the placenta essential for fetal development and survival as mis-localization was found to impair embryonic growth and induce fetal death (21,22) - Found at increased levels in preeclamptic patients (23) - Expressed in newly formed decidual cells and inhibition decelerated proliferation and differentiation of stromal cells (24) - Inhibition impaired angiogenesis and decreased neovascularization of endometrial tissue (25) - High expression induced hypoxia in hepatocellular carcinoma cells while its knockdown was able to reverse these effects (26) - Increased expression of immunosuppressive chemokines and factors (i.e., CCL2, CXCR2, CXCR4, CCL20, CXCL8, Foxp3, TGF-$\beta$, IL-10) (27,28) |
|  | **APOA1** | p = 2.1E-02  ($\uparrow$ CTL, $\downarrow$ KO) | - Elevated in the plasma and placental tissues of women with preeclampsia and early miscarriage (29,30) - siRNA knockdown increased the proliferation and invasion of HTR8/SVneo trophoblast cells while recombinant Apoa1 reversed these effects (29) - Overexpression attenuated endothelial cell migration and tube formation by inactivating Erk1/2 and suppressing Pigf expression (31) - Another study found that Apoa1 increased the proliferation, branching, and tubal formation of endothelial progenitor cells (32) - Upregulated endothelial NO production and inhibited vasoconstriction induced by thromboxane A1 receptor agonist (33) - Attenuated inflammation by decreasing neutrophil activation and spreading (34) |
|  | **CA2** | p = 3.7E-02  ($\uparrow$ CTL, $\downarrow$ KO) | - Normally increased in the placental villous endothelium as pregnancy progresses to mediate bicarbonate/CO_2_ removal (35) - Also believed to be decreased in the basolateral plasma membrane and decidual glands post-implantation to promote an acidic intrauterine environment conducive for decidualization (36) - Inhibition prevented experimentally induced abortion (36) - Anti-CA II antibody levels were detected in cases of preeclampsia and recurrent pregnancy loss (37,38) - Knockdown decreased survival of tumor endothelial cells and reduced *in vivo* tumor angiogenesis (39) - Could be an indicator for hypoxia as low O_2_ conditions increased the expression of CA2 in chondrocytes (40) |
|  | **VIM** | p = 3.9E-02  ($\uparrow$ CTL, $\downarrow$ KO) | - Degradation impaired the invasion and migration of HTR8/SVneo trophoblasts *in vitro* (41) - Increased in the chorionic villi and decidual cells of placentas from women with placenta previa (42) - Could be a marker for vascular trophoblast giant cells in mice since it exhibited strong expression and colocalization with Vegf and Flt1 during trophoblast giant cell differentiation at E7.5 (43) - Increased in the placentas of women with HELLP syndrome (44) - Downregulation believed to mediate IUGR in pregnant mice exposed to cholecalciferol by impairing migration and invasion of trophoblasts (45) - Knockdown inhibited tube formation of HUVEC endothelial cells by interfering with binding of proangiogenic peptide SP (46) - Knockout in kidneys increased production and sensitivity to vasoconstrictor endothelin in mice and decreased synthesis and responsiveness to NO (47) - Found it could be anti-inflammatory by regulating cytokine release (i.e., IFN-I, IL-6, IL-12) via TBK1-IKK$\varepsilon$-IRF3 axis (48) - Increased M2 macrophages (49) - Other studies found it promoted inflammation and maturation of IL-1$\beta$ by activating NLRP3 inflammasome (50) |
|  | **PTGS1** | p = 1.9E-02  ($\uparrow$ CTL, $\downarrow$ KO) | - Increased in the placental bed of preeclamptic patients (51) - Prostaglandins have been shown to increase adhesiveness of extravillous trophoblasts by upregulating integrin and focal adhesion kinase levels (52) - Could be an indicator for IUGR as surgically inducing IUGR in guinea pigs upregulated of PTGS1 expression in the myometrium (53) - Inhibition suppressed angiogenic processes, such as tube formation *in vitro* (54) - Endothelial-specific deletion reduced cellular response to vasodilators like acetylcholine and phenylephrine (55) - Can induce endothelial cell inflammation (56) - Involved in M2 macrophage polarization via IL-4 (57) |
|  | **KNG1** | p = 8.4E-03  ($\uparrow$ CTL, $\downarrow$ KO) | - Increased in the muscles of pig fetuses with IUGR at gestational day 60 as an indicator of impaired muscle development (58) - Overexpression impaired generation of blood vessels, tube formation, and expression of proangiogenic factor VEGF (59) - Associated with increased vasodilation as a precursor for the vasodilator factor bradykinin (60) - Found to be proinflammatory as depletion decreased IL-1$\beta$ and IFN$\gamma$ levels during viral infection (61) - Proteolytic cleavage and release of bradykinin could induce proinflammatory response by NO and prostaglandins and through recruitment of neutrophils (62,63) |
|  | **LRP1** | p = 2.1E-02  ($\downarrow$ CTL, $\uparrow$ KO) | - Depletion in endothelial cells increased their proliferation and the number of angiogenic sprouts in mouse retinas (64) - Another study found that loss of LRP1 in mice actually led to lethal vascular defects due to impairments in the S1P and PDGF-BB signaling pathways (65) - Could be a marker for hypoxia since hypoxic conditions induced LRP1 expression in vascular smooth muscle cells (66) - Deficiency in murine aortic rings attenuated reactivity to numerous vasoconstrictors (i.e., phenylephrine, K^+^, U-46619) (67) - Found to suppress inflammation via NF$\kappa$B, Akt/mTOR, and TLR signaling (68,69) - Deficiency in myeloid cells and macrophages found to increase production of proinflammatory IL-1$\beta$, IL-6, and TNF-$\alpha$ (69) - Found to be important for phagocytosis/efferocytosis in macrophages (70) |
|  | **TGM1** | p = 2.9E-02  ($\downarrow$ CTL, $\uparrow$ KO) | - Could be a marker for hypoxia as exposure of human small airway epithelial cells to hypoxic conditions significantly upregulated TGM1 levels (71) - Depletion increased expression of cytokines and chemokines like IL-1$\beta$, CXCL1, CXCL2, and CCL2 (72) - Significantly correlated with the infiltration of neutrophils (73) |
|  | **SLC44A1** | p = 5.6E-03  ($\downarrow$ CTL, $\uparrow$ KO) | - Expressed by syncytial trophoblasts and the fetal endothelium of the chorionic villi from 6 weeks of gestation to term (74,75) - Acts as a transporter by which trophoblasts and endothelial cells can regulate choline uptake during pregnancy to provide substrate for membrane lipids, acetylcholine and methylation reactions (76) - Involved in NLRP3 inflammasome activation and production of proinflammatory cytokines (i.e., IL-1$\beta$, IL-18) in macrophages (77,78) |
|  | **FLOT1** | p = 2.4E-02  ($\downarrow$ CTL, $\uparrow$ KO) | - Found it be immunosuppressive as its depletion activates STING pathway and production of chemokines like CCL5 and CXCL10 (79) - Involved in migration of neutrophils (80) |
|  | **CTSK** | p = 3.3E-02  ($\downarrow$ CTL, $\uparrow$ KO) | - Knockout in mice resulted in a lower body weight due to reduced white adipose tissue from increased lipolysis and use of fatty acids (81) - Deficiency in mice was also impaired endothelial cell invasion, proliferation, tube formation, and the expression of proangiogenic factors (i.e., Notch1, Vegf, Flt-1, p-Akt) (82) - Induced rapid production of various kinins involved in vasodilation in macrophages (i.e., BK(5-9), Lys-bradykinin) by cleaving the Gly384-Phe385 bond (83) - Promoted inflammation by increasing expression of proinflammatory genes (i.e., TLR4, TLR5, TLR9) and cytokines (i.e., TNF-$\alpha$, IL-6, IL-1$\alpha$, IL-17) (84,85) - Positively linked with macrophage and neutrophil infiltration as well as M2 macrophage polarization (85–87) |
|  | **FAM3D** | p = 3.8E-04  ($\uparrow$ CTL, $\downarrow$ KO) | - Deficiency in mice reduced angiotensin II-induced hypertension by preventing uncoupling of endothelial nitric oxide synthase (eNOS) to promote vasorelaxation (88) - Found it could alter the migration and infiltration of neutrophils and other leukocytes (89,90) - Depletion increased proinflammatory cytokines (i.e., IL-1, CXCL1) (91) |
|  | **ATP7A** | p = 3.6E-02  ($\uparrow$ CTL, $\downarrow$ KO) | - Expressed on the fetal side of syncytiotrophoblasts in mice and was found to increase copper levels in pups when overexpressed, which suggested an essential role in copper ion transport during gestation (92) - Mice with an enterocyte-specific knockout of Atp7a exhibited growth impairment, neurological deterioration, and early postnatal mortality (93) - Could be a marker for hypoxia since an upregulation of *Atp7a* was observed in rat intestinal epithelial cells exposed to hypoxic conditions (94) - Deficiency or dysfunction in murine endothelial cells impaired neovascularization in mice by degrading Vegfr2 (95) - Exhibited a protective effect against angiotensin II-induced vasoconstriction by binding with copper-dependent transcription factor Atox1 to promote SOD3 expression, which inhibits angiotensin II activity (96) - Found to limit copper-dependent proinflammation as dysfunction resulted in increased number of proinflammatory cells (Mac3^+^, MCP-1^+^, CD45^+^ cells) and cytokines (TNF$\alpha$, IL-1$\beta$, MCP-1, IL-6) (97) - Involved in macrophage bactericidal activity (98) |
|  | **ESD** | p = 2.8E-04  ($\downarrow$ CTL, $\uparrow$ KO) | - Found to enhance IFN-I signaling by activating IRF3 or upstream factors (99) |
|  | **HSPA2** | p = 2.4E-02  ($\downarrow$ CTL, $\uparrow$ KO) | - Could be a marker for tissue hypoxia as under hypoxic conditions HSPA2 expression was inhibited in keratinocytes (100) - Another study found that hypoxic conditions increased HSPA2 in hepatocellular carcinoma cells due to increased HIF-1$\alpha$ binding to the hypoxic response element (HRE1) present on the HSPA2 promoter (101) |
|  | **PALM** | p = 4.1E-02  ($\downarrow$ CTL, $\uparrow$ KO) | - Repressed migration and angiogenic formation of tube-like structures of lymphatic endothelial cells by modulating cell-substrate adhesion, filopodia formation and plasma membrane blebbing (102) |
|  | **WASF2** | p = 2.5E-02  ($\downarrow$ CTL, $\uparrow$ KO) | - Oxidative stress in HTR-8/SVneo trophoblasts decreased cell proliferation, increased apoptosis, and reduced invasion by depleting Wasf2 (103) - Decreased in preeclamptic placentas where it is inversely correlated with oxidative stress levels (103) - Deficiency decreased sprouting and branching of endothelial cells in mice by impairing actin reorganization, which also resulted in lethality by E10 (104) - Another study found that its repression increased HUVEC lamellipodia formation *in vitro* to promote angiogenesis (105) |
|  | **APH1A** | p = 2.4E-02  ($\downarrow$ CTL, $\uparrow$ KO) | - Knockout in mouse embryos resulted in a lethal phenotype where vascularization of the yolk sac was nearly absent by E10.5 (106) |
|  | **ANXA8** | p = 4.1E-02  ($\downarrow$ CTL, $\uparrow$ KO) | - Deficiency in HUVECs impaired angiogenic sprouting due to defective formation of CD63/VEGFR2/Integrin $\beta$1 complex, which is necessary for the VEGF-A transduction pathway (107) |
|  | **TGFB2** | p = 4.7E-02  ($\downarrow$ CTL, $\uparrow$ KO) | - Inhibition increased the invasiveness of extra villous trophoblasts while addition of exogenous Tgfb2 reversed these effects by reducing MMP9 (108) - Involved in the differentiation of endovascular extra villous trophoblasts as recombinant Tgfb2 downregulated the expression key enEVT markers (i.e., Cdh5, Mmp-1, Il-1b, Il-8) (109) - Impaired spiral artery remodeling by inhibiting HTR8/SVneo trophoblast network formation *in vitro* (109) - Vector promoted migration and tube formation ability of endothelial cells (110) - Could be a hypoxia marker since HUVECs exposed to hypoxic conditions exhibit upregulated Tgfb2 levels (111) - Induced vasoconstriction in kidneys of mice and increased levels of vasoconstrictors endothelin 1 and angiotensin II (112) - Positively correlated with macrophage infiltration and M1 macrophage marker PTGS2 (113,114) |
|  | **ZDHHC21** | p = 3.0E-02  ($\downarrow$ CTL, $\uparrow$ KO) | - Deficiency in mice reduced vasoconstriction of the renal arteries while upregulation impaired renal perfusion (115) - Abrogation in mice also impaired their responsiveness to the vasoconstriction factor phenylephrine and induced hypotension (116) |
|  | **GLB1** | p = 3.4E-02  ($\downarrow$ CTL, $\uparrow$ KO) | - Reported case of G1M gangliosidosis and IUGR caused by homozygous splicing mutation of *GLB1* gene, which resulted in lysosomal storage defects and diffuse vacuolization of cyto- and syncytiotrophoblasts, stromal cells, and amniocytes in the placenta (117) - Genetic knockdown increased NO production and endothelial nitric oxide synthase levels to promote vasodilation (118) |
|  | **MAN2A2** | p = 2.0E-02  ($\downarrow$ CTL, $\uparrow$ KO) | - Could be a marker for hypoxia as exposure to hypobaric hypoxia downregulated Man2a2 levels in rats (119) |
|  | **TSPAN8** | p = 7.6E-03  ($\uparrow$ CTL, $\downarrow$ KO) | - Knockdown in mice impaired migration and sprouting of endothelial cells due to the decreased expression of proangiogenic CD31 and VEGFR2 (120) |
|  | **SUN2** | p = 4.5E-02  ($\downarrow$ CTL, $\uparrow$ KO) | - Depletion altered the spreading and actomyosin/RhoA activity of vascular smooth muscle cells (121) |
|  | **ATRNL1** | p = 2.4E-02  ($\downarrow$ CTL, $\uparrow$ KO) | - Overexpression upregulated the proliferation, migration, and invasion of Ishikawa endometrial cells while silencing conferred opposite effects by altering EMT processes (122,123) |
|  | **SRD5A1** | p = 1.7E-02  ($\uparrow$ CTL, $\downarrow$ KO) | - Deficiency impaired decidualization, compromised vessel permeability, and a downregulated angiogenic factors in mice (124) |
|  | **PRL4A1** | p = 4.2E-02  ($\uparrow$ CTL, $\downarrow$ KO) | - Characterized as a trophoblast giant cell marker involved in invasion (125,126) |
| MicroRNAs | **miR-362-5p** | p = 4.15E-10  ($\uparrow$ CTL, $\downarrow$ KO) | - Promoted the proliferation and inhibited the apoptosis of trophoblast cells by targeting glutathione-disulfide reductase (127) - miR-362-5p expression has been shown to be downregulated in GDM placenta tissues (127) |
|  | **miR-541-5p** | p = 3.60E-06  ($\uparrow$ CTL, $\downarrow$ KO) | - Significantly downregulated in preeclamptic pregnancies and mediates trophoblast invasion by regulating FSTL3 levels (128,129) - Found to induce M2 macrophage polarization by activating the JAK2/STAT3 pathway activation (130) |
|  | **miR-30a-3p** | p = 6.19E-06  ($\uparrow$ CTL, $\downarrow$ KO) | - Upregulated in preeclamptic placental samples where it was found to reduce trophoblast invasion and promote apoptosis through its effects on IGF-1 (131) - Also found to enhance the proliferation and migration of HTR-8/SVneo trophoblast cells when inhibited (132) - Has been found to inhibit the expression of HIF2A transcripts, thus could play roles in mediating hypoxia (133) - Inhibited decidua-derived MSC proliferation, and induced their apoptosis when upregulated (134) - Suppressed HUVEC tube formation when exposed to the supernatant from miR-30a-transfected dMSCs (134) |
|  | **miR-182-3p** | p = 2.00E-03  ($\uparrow$ CTL, $\downarrow$ KO) | - Upregulated in EVs from GDM and preeclampsia plasma samples (135,136) |
|  | **miR-361-3p** | p = 1.61E-04  ($\uparrow$ CTL, $\downarrow$ KO) | - Downregulated in the blood samples of unexplained recurrent pregnancy loss (RPL) patients (137) - Believed to normally control apoptosis within the reproductive tract by decreasing BCL-2 levels (137) |
|  | **miR-149-5p** | p = 1.00E-03  ($\downarrow$ CTL, $\uparrow$ KO) | - Impaired decidualization of T-HESCs by promoting cell apoptosis through negative regulation of PARP-2 but positive regulation of caspase-8 levels (138) - Downregulated in the preeclampsia placental samples where it was believed to normally promote trophoblast proliferation and invasion by reducing expression of various targets like ENG, ERP44, or TFPI2 (139–141) - Promoted the angiogenesis of HUVEC cells by downregulating ERP44 (140) - Inhibition of miR-149 believed to protect against recurrent miscarriage by upregulating RUNX2 and activating the PTEN/Akt signaling pathway (142) - Inhibited proinflammatory cytokine production in osteoarthritis and rheumatoid arthritis synovial fibroblasts (143) |
|  | **miR-181a-1-3p** | p = 4.83E-05  ($\uparrow$ CTL, $\downarrow$ KO) | - Inhibited macrophage activation in chronic liver injury (144) - Shown to be an essential regulator of inflammation in macrophages and dendritic cells (145) - Also inhibited vascular inflammation by targeting TAB2 and NEMO, which regulates the NF-κB signaling pathway (146) |
|  | **miR-23a-3p** | p = 3.25E-14  ($\downarrow$ CTL, $\uparrow$ KO) | - Found to induce endothelial progenitor cells (EPCs) loss and impair endothelial function of the differentiated progeny (147) - Knockdown in HUVECs promoted cell proliferation, migration, and tube formation (148) |
|  | **miR-30c-5p** | p = 2.13E-09  ($\downarrow$ CTL, $\uparrow$ KO) | - Increased in placental HUVECs from women with GDM (149) - Downregulation found to promote release of pro-inflammatory factors like IL-1$\beta$ and pro-apoptotic signals like caspase-3, which impaired endothelial healing *in vivo* (150) |
|  | **miR-450a-5p** | p = 8.87E-09  ($\uparrow$ CTL, $\downarrow$ KO) | - Believed to promote syncytial differentiation as miR-450a-5p was upregulated in EVs from pregnant mice and those from the conditioned media of syncytial-like trophoblasts (151) |

**Table References**

1. Fitzgerald HC, Kelleher AM, Ranjit C, Schust DJ, Spencer TE. Basolateral secretions of human endometrial epithelial organoids impact stromal cell decidualization. Mol Hum Reprod. 2023 Apr 3;29(4).

2. Yu N, Chen X, Du M, Li H, Wang Y, Jiang F, et al. Long Non-Coding RNA ZEB2-AS1 Augments Activity of Trophoblast Cells and Prevents the Development of Recurrent Spontaneous Abortion in Mice Through EZH2-Mediated CST3 Inhibition. Reprod Sci Thousand Oaks Calif. 2022 Mar;29(3):963–74.

3. Zou J, Chen Z, Wei X, Chen Z, Fu Y, Yang X, et al. Cystatin C as a potential therapeutic mediator against Parkinson’s disease via VEGF-induced angiogenesis and enhanced neuronal autophagy in neurovascular units. Cell Death Dis. 2017 Jun 1;8(6):e2854.

4. Frendéus KH, Wallin H, Janciauskiene S, Abrahamson M. Macrophage responses to interferon-gamma are dependent on cystatin C levels. Int J Biochem Cell Biol. 2009 Nov;41(11):2262–9.

5. Xu L, Li YH, Zhao WJ, Sang YF, Chen JJ, Li DJ, et al. RhoB Promotes Endometrial Stromal Cells Decidualization Via Semaphorin3A/PlexinA4 Signaling in Early Pregnancy. Endocrinology. 2022 Oct 11;163(11):bqac134.

6. Han J, Yang BP, Li YL, Li HM, Zheng XH, Yu LL, et al. RhoB/ROCK mediates oxygen-glucose deprivation-stimulated syncytiotrophoblast microparticle shedding in preeclampsia. Cell Tissue Res. 2016 Nov;366(2):411–25.

7. Adini I, Rabinovitz I, Sun JF, Prendergast GC, Benjamin LE. RhoB controls Akt trafficking and stage-specific survival of endothelial cells during vascular development. Genes Dev. 2003 Nov 1;17(21):2721–32.

8. Sabatel C, Malvaux L, Bovy N, Deroanne C, Lambert V, Gonzalez MLA, et al. MicroRNA-21 exhibits antiangiogenic function by targeting RhoB expression in endothelial cells. PloS One. 2011 Feb 10;6(2):e16979.

9. Wojciak-Stothard B, Zhao L, Oliver E, Dubois O, Wu Y, Kardassis D, et al. Role of RhoB in the regulation of pulmonary endothelial and smooth muscle cell responses to hypoxia. Circ Res. 2012 May 25;110(11):1423–34.

10. Huang G, Su J, Zhang M, Jin Y, Wang Y, Zhou P, et al. RhoB regulates the function of macrophages in the hypoxia-induced inflammatory response. Cell Mol Immunol. 2017 Mar;14(3):265–75.

11. Yu H, Pan J, Zheng S, Cai D, Luo A, Xia Z, et al. Hepatocellular Carcinoma Cell-Derived Exosomal miR-21-5p Induces Macrophage M2 Polarization by Targeting RhoB. Int J Mol Sci. 2023 Feb 27;24(5).

12. Beharier O, Tyurin VA, Goff JP, Guerrero-Santoro J, Kajiwara K, Chu T, et al. PLA2G6 guards placental trophoblasts against ferroptotic injury. Proc Natl Acad Sci U S A. 2020 Nov 3;117(44):27319–28.

13. Zhang Y, Yang Y, Chen W, Mi C, Xu X, Shen Y, et al. BaP/BPDE suppressed endothelial cell angiogenesis to induce miscarriage by promoting MARCHF1/GPX4-mediated ferroptosis. Environ Int. 2023 Oct;180:108237.

14. Xu X, Zhu M, Zu Y, Wang G, Li X, Yan J. Nox2 inhibition reduces trophoblast ferroptosis in preeclampsia via the STAT3/GPX4 pathway. Life Sci. 2024 Apr 15;343:122555.

15. Mistry HD, Kurlak LO, Williams PJ, Ramsay MM, Symonds ME, Broughton Pipkin F. Differential expression and distribution of placental glutathione peroxidases 1, 3 and 4 in normal and preeclamptic pregnancy. Placenta. 2010 May;31(5):401–8.

16. Li P, Jiang M, Li K, Li H, Zhou Y, Xiao X, et al. Glutathione peroxidase 4-regulated neutrophil ferroptosis induces systemic autoimmunity. Nat Immunol. 2021 Sep;22(9):1107–17.

17. Amaral EP, Foreman TW, Namasivayam S, Hilligan KL, Kauffman KD, Barbosa Bomfim CC, et al. GPX4 regulates cellular necrosis and host resistance in Mycobacterium tuberculosis infection. J Exp Med. 2022 Nov 7;219(11).

18. Xu C, Sun S, Johnson T, Qi R, Zhang S, Zhang J, et al. The glutathione peroxidase Gpx4 prevents lipid peroxidation and ferroptosis to sustain Treg cell activation and suppression of antitumor immunity. Cell Rep. 2021 Jun 15;35(11):109235.

19. Nagai A, Takebe K, Nio-Kobayashi J, Takahashi-Iwanaga H, Iwanaga T. Cellular Expression of the Monocarboxylate Transporter (MCT) Family in the Placenta of Mice. Placenta. 2010 Feb 1;31(2):126–33.

20. Settle P, Mynett K, Speake P, Champion E, Doughty IM, Sibley CP, et al. Polarized lactate transporter activity and expression in the syncytiotrophoblast of the term human placenta. Placenta. 2004 Jul;25(6):496–504.

21. Liang X, Tang S, Li D, Song Y, He M, Duan Y, et al. Shoutai Wan Improves Embryo Survival by Regulating Aerobic Glycolysis of Trophoblast Cells in a Mouse Model of Recurrent Spontaneous Abortion. Evid-Based Complement Altern Med ECAM. 2022;2022:8251503.

22. Moreau JLM, Artap ST, Shi H, Chapman G, Leone G, Sparrow DB, et al. Cited2 is required in trophoblasts for correct placental capillary patterning. Dev Biol. 2014 Aug 1;392(1):62–79.

23. Kay HH, Zhu S, Tsoi S. Hypoxia and lactate production in trophoblast cells. Placenta. 2007 Sep;28(8–9):854–60.

24. Zuo RJ, Gu XW, Qi QR, Wang TS, Zhao XY, Liu JL, et al. Warburg-like Glycolysis and Lactate Shuttle in Mouse Decidua during Early Pregnancy. J Biol Chem. 2015 Aug 28;290(35):21280–91.

25. Bahrami A, Ayen E, Razi M, Behfar M. Effects of atorvastatin and resveratrol against the experimental endometriosis; evidence for glucose and monocarboxylate transporters, neoangiogenesis. Life Sci. 2021 May 1;272:119230.

26. Shen J, Wu Z, Zhou Y, Yang D, Wang X, Yu B, et al. Knockdown of SLC16A3 decreases extracellular lactate concentration in hepatocellular carcinoma, alleviates hypoxia and induces ferroptosis. Biochem Biophys Res Commun. 2024 Nov 12;733:150709.

27. Tao Q, Li X, Zhu T, Ge X, Gong S, Guo J, et al. Lactate Transporter SLC16A3 (MCT4) as an Onco-Immunological Biomarker Associating Tumor Microenvironment and Immune Responses in Lung Cancer. Int J Gen Med. 2022;15:4465–74.

28. Zhu T, Ge X, Gong S, Guo S, Tao Q, Guo J, et al. Prognostic value of lactate transporter SLC16A1 and SLC16A3 as oncoimmunological biomarkers associating tumor metabolism and immune evasion in glioma. Cancer Innov. 2022 Oct;1(3):229–39.

29. Liu Z, Pei J, Zhang X, Wang C, Tang Y, Liu H, et al. APOA1 Is a Novel Marker for Preeclampsia. Int J Mol Sci. 2023 Nov 15;24(22).

30. Verma P, Nair RR, Singh S, Rajender S, Khanna A, Jha RK, et al. High Level of APOA1 in Blood and Maternal Fetal Interface Is Associated With Early Miscarriage. Reprod Sci Thousand Oaks Calif. 2019 May;26(5):649–56.

31. Hu J, Chen ZT, Su KY, Lian Y, Lu L, Hu ADN. Apolipoprotein A1 suppresses the hypoxia-induced angiogenesis of human retinal endothelial cells by targeting PlGF. Int J Ophthalmol. 2023;16(1):33–9.

32. González-Pecchi V, Valdés S, Pons V, Honorato P, Martinez LO, Lamperti L, et al. Apolipoprotein A-I enhances proliferation of human endothelial progenitor cells and promotes angiogenesis through the cell surface ATP synthase. Microvasc Res. 2015 Mar;98:9–15.

33. Cabou C, Honorato P, Briceño L, Ghezali L, Duparc T, León M, et al. Pharmacological inhibition of the F(1) -ATPase/P2Y(1) pathway suppresses the effect of apolipoprotein A1 on endothelial nitric oxide synthesis and vasorelaxation. Acta Physiol Oxf Engl. 2019 Jul;226(3):e13268.

34. Murphy AJ, Woollard KJ, Suhartoyo A, Stirzaker RA, Shaw J, Sviridov D, et al. Neutrophil activation is attenuated by high-density lipoprotein and apolipoprotein A-I in in vitro and in vivo models of inflammation. Arterioscler Thromb Vasc Biol. 2011 Jun;31(6):1333–41.

35. Mühlhauser J, Crescimanno C, Rajaniemi H, Parkkila S, Milovanov AP, Castellucci M, et al. Immunohistochemistry of carbonic anhydrase in human placenta and fetal membranes. Histochemistry. 1994 Feb;101(2):91–8.

36. Chiang WL, Liu JY, Liao CY, Yang SF, Hsieh YS, Chu SC. Alternation of cytosolic carbonic anhydrase isoenzymes during deciduomatal development in pregnant mice. Fertil Steril. 2004 Oct;82 Suppl 3:1095–100.

37. Aliyazicioglu R, Guven S, Mentese A, Kolayli S, Cengiz S, Deger O, et al. Serum anti-carbonic anhydrase II antibodies and oxidant-antioxidant balance in pre-eclampsia. Am J Reprod Immunol N Y N 1989. 2011 Oct;66(4):297–303.

38. Karahan SC, Guven S, Mentese A, Bacak A, Kopuz M, Ozeren M. Serum anti-carbonic anhydrase I and II antibodies and idiopathic recurrent pregnancy loss. Reprod Biomed Online. 2009 Dec 1;19(6):859–63.

39. Annan DA, Maishi N, Soga T, Dawood R, Li C, Kikuchi H, et al. Carbonic anhydrase 2 (CAII) supports tumor blood endothelial cell survival under lactic acidosis in the tumor microenvironment. Cell Commun Signal CCS. 2019 Dec 17;17(1):169.

40. Yan M, Cai L, Duan X, Tycksen ED, Rai MF. Carbonic anhydrase 2 is important for articular chondrocyte function and metabolic homeostasis. Bone. 2025 Jan;190:117313.

41. Xiong L, Ye X, Chen Z, Fu H, Li S, Xu P, et al. Advanced Maternal Age-associated SIRT1 Deficiency Compromises Trophoblast Epithelial-Mesenchymal Transition through an Increase in Vimentin Acetylation. Aging Cell. 2021 Oct;20(10):e13491.

42. Soyama H, Miyamoto M, Ishibashi H, Iwahashi H, Matsuura H, Kakimoto S, et al. Placenta previa may acquire invasive nature by factors associated with epithelial-mesenchymal transition and matrix metalloproteinases. J Obstet Gynaecol Res. 2020 Dec;46(12):2526–33.

43. Scherholz PLA, de Souza PC, Spadacci-Morena DD, Katz SG. Vimentin is synthesized by mouse vascular trophoblast giant cells from embryonic day 7.5 onwards and is a characteristic factor of these cells. Placenta. 2013 Jul;34(7):518–25.

44. Sak ME, Deveci E, Turgut A, Sak S, Evsen MS, Gul T, et al. Placental expression of vimentin, desmin and ultrastructural changes in the villi in patients with HELLP syndrome. Eur Rev Med Pharmacol Sci. 2013 Apr;17(7):874–8.

45. Ma L, Chen YH, Liu ZB, Gao L, Wang B, Fu L, et al. Supplementation with high-dose cholecalciferol throughout pregnancy induces fetal growth restriction through inhibiting placental proliferation and trophoblast epithelial-mesenchymal transition. J Nutr Biochem. 2021 May;91:108601.

46. Glaser-Gabay L, Raiter A, Battler A, Hardy B. Endothelial cell surface vimentin binding peptide induces angiogenesis under hypoxic/ischemic conditions. Microvasc Res. 2011 Nov;82(3):221–6.

47. Terzi F, Henrion D, Colucci-Guyon E, Federici P, Babinet C, Levy BI, et al. Reduction of renal mass is lethal in mice lacking vimentin. Role of endothelin-nitric oxide imbalance. J Clin Invest. 1997 Sep 15;100(6):1520–8.

48. Liu H, Ye G, Liu X, Xue M, Zhou Q, Zhang L, et al. Vimentin inhibits type I interferon production by disrupting the TBK1-IKKε-IRF3 axis. Cell Rep. 2022 Oct 11;41(2):111469.

49. Yu MB, Guerra J, Firek A, Langridge WHR. Extracellular vimentin modulates human dendritic cell activation. Mol Immunol. 2018 Dec;104:37–46.

50. dos Santos G, Rogel MR, Baker MA, Troken JR, Urich D, Morales-Nebreda L, et al. Vimentin regulates activation of the NLRP3 inflammasome. Nat Commun. 2015 Mar 12;6:6574.

51. Wetzka B, Nüsing R, Charnock-Jones DS, Schäfer W, Zahradnik HP, Smith SK. Cyclooxygenase-1 and -2 in human placenta and placental bed after normal and pre-eclamptic pregnancies. Hum Reprod Oxf Engl. 1997 Oct;12(10):2313–20.

52. Nayeem SB, Dharmarajan A, Keelan JA. Paracrine communication modulates production of Wnt antagonists and COX1-mediated prostaglandins in a decidual-trophoblast co-culture model. Mol Cell Endocrinol. 2015 Apr 15;405:52–62.

53. Palliser HK, Kelleher MA, Welsh TN, Zakar T, Hirst JJ. Mechanisms leading to increased risk of preterm birth in growth-restricted guinea pig pregnancies. Reprod Sci Thousand Oaks Calif. 2014 Feb;21(2):269–76.

54. Tsujii M, Kawano S, Tsuji S, Sawaoka H, Hori M, DuBois RN. Cyclooxygenase regulates angiogenesis induced by colon cancer cells. Cell. 1998 May 29;93(5):705–16.

55. Mitchell JA, Shala F, Pires MEL, Loy RY, Ravendren A, Benson J, et al. Endothelial cyclooxygenase-1 paradoxically drives local vasoconstriction and atherogenesis despite underpinning prostacyclin generation. Sci Adv. 2021 Mar;7(12).

56. Garshick MS, Tawil M, Barrett TJ, Salud-Gnilo CM, Eppler M, Lee A, et al. Activated Platelets Induce Endothelial Cell Inflammatory Response in Psoriasis via COX-1. Arterioscler Thromb Vasc Biol. 2020 May;40(5):1340–51.

57. Shay AE, Diwakar BT, Guan BJ, Narayan V, Urban JFJ, Prabhu KS. IL-4 up-regulates cyclooxygenase-1 expression in macrophages. J Biol Chem. 2017 Sep 1;292(35):14544–55.

58. Cortes-Araya Y, Cheung S, Ho W, Stenhouse C, Ashworth CJ, Esteves CL, et al. Effects of foetal size, sex and developmental stage on adaptive transcriptional responses of skeletal muscle to intrauterine growth restriction in pigs. Sci Rep. 2024 Apr 11;14(1):8500.

59. Xu J, Fang J, Cheng Z, Fan L, Hu W, Zhou F, et al. Overexpression of the Kininogen-1 inhibits proliferation and induces apoptosis of glioma cells. J Exp Clin Cancer Res CR. 2018 Aug 2;37(1):180.

60. Legchenko E, Chouvarine P, Qadri F, Specker E, Nazaré M, Wesolowski R, et al. Novel Tryptophan Hydroxylase Inhibitor TPT-001 Reverses PAH, Vascular Remodeling, and Proliferative-Proinflammatory Gene Expression. JACC Basic Transl Sci. 2024 Jul;9(7):890–902.

61. Köhler J, Maletzki C, Koczan D, Frank M, Springer A, Steffen C, et al. Kininogen supports inflammation and bacterial spreading during Streptococccus Pyogenes Sepsis. EBioMedicine. 2020 Aug;58:102908.

62. Paegelow I, Trzeczak S, Böckmann S, Vietinghoff G. Migratory responses of polymorphonuclear leukocytes to kinin peptides. Pharmacology. 2002 Nov;66(3):153–61.

63. Sato E, Koyama S, Nomura H, Kubo K, Sekiguchi M. Bradykinin stimulates alveolar macrophages to release neutrophil, monocyte, and eosinophil chemotactic activity. J Immunol Baltim Md 1950. 1996 Oct 1;157(7):3122–9.

64. Mao H, Lockyer P, Townley-Tilson WHD, Xie L, Pi X. LRP1 Regulates Retinal Angiogenesis by Inhibiting PARP-1 Activity and Endothelial Cell Proliferation. Arterioscler Thromb Vasc Biol. 2016 Feb;36(2):350–60.

65. Nakajima C, Haffner P, Goerke SM, Zurhove K, Adelmann G, Frotscher M, et al. The lipoprotein receptor LRP1 modulates sphingosine-1-phosphate signaling and is essential for vascular development. Dev Camb Engl. 2014 Dec;141(23):4513–25.

66. Castellano J, Aledo R, Sendra J, Costales P, Juan-Babot O, Badimon L, et al. Hypoxia stimulates low-density lipoprotein receptor-related protein-1 expression through hypoxia-inducible factor-1α in human vascular smooth muscle cells. Arterioscler Thromb Vasc Biol. 2011 Jun;31(6):1411–20.

67. LRP1 (Low-Density Lipoprotein Receptor-Related Protein 1) Regulates Smooth Muscle Contractility by Modulating Ca(2+) Signaling and Expression of Cytoskeleton-Related Proteins. Vol. 38. United States; 2018.

68. Luo L, Wall AA, Tong SJ, Hung Y, Xiao Z, Tarique AA, et al. TLR Crosstalk Activates LRP1 to Recruit Rab8a and PI3Kγ for Suppression of Inflammatory Responses. Cell Rep. 2018 Sep 11;24(11):3033–44.

69. Mantuano E, Brifault C, Lam MS, Azmoon P, Gilder AS, Gonias SL. LDL receptor-related protein-1 regulates NFκB and microRNA-155 in macrophages to control the inflammatory response. Proc Natl Acad Sci U S A. 2016 Feb 2;113(5):1369–74.

70. Yancey PG, Blakemore J, Ding L, Fan D, Overton CD, Zhang Y, et al. Macrophage LRP-1 controls plaque cellularity by regulating efferocytosis and Akt activation. Arterioscler Thromb Vasc Biol. 2010 Apr;30(4):787–95.

71. Li L, Watson CJ, Dubourd M, Bruton A, Xu M, Cooke G, et al. HIF-1-Dependent TGM1 Expression is Associated with Maintenance of Airway Epithelial Junction Proteins. Lung. 2016 Oct;194(5):829–38.

72. Haneda T, Imai Y, Uchiyama R, Jitsukawa O, Yamanishi K. Activation of Molecular Signatures for Antimicrobial and Innate Defense Responses in Skin with Transglutaminase 1 Deficiency. PloS One. 2016;11(7):e0159673.

73. Wu R, Li D, Zhang S, Wang J, Chen K, Tuo Z, et al. A pan-cancer analysis of the oncogenic and immunological roles of transglutaminase 1 (TGM1) in human cancer. J Cancer Res Clin Oncol. 2024 Mar 12;150(3):123.

74. Baumgartner HK, Trinder KM, Galimanis CE, Post A, Phang T, Ross RG, et al. Characterization of choline transporters in the human placenta over gestation. Placenta. 2015 Dec;36(12):1362–9.

75. Iwao B, Yara M, Hara N, Kawai Y, Yamanaka T, Nishihara H, et al. Functional expression of choline transporter like-protein 1 (CTL1) and CTL2 in human brain microvascular endothelial cells. Neurochem Int. 2016 Feb;93:40–50.

76. Yara M, Iwao B, Hara N, Yamanaka T, Uchino H, Inazu M. Molecular and functional characterization of choline transporter in the human trophoblastic cell line JEG-3 cells. Placenta. 2015 Jun;36(6):631–7.

77. Sanchez-Lopez E, Zhong Z, Stubelius A, Sweeney SR, Booshehri LM, Antonucci L, et al. Choline Uptake and Metabolism Modulate Macrophage IL-1β and IL-18 Production. Cell Metab. 2019 Jun 4;29(6):1350-1362.e7.

78. Snider SA, Margison KD, Ghorbani P, LeBlond ND, O’Dwyer C, Nunes JRC, et al. Choline transport links macrophage phospholipid metabolism and inflammation. J Biol Chem. 2018 Jul 20;293(29):11600–11.

79. Wang Y, Meng L, Meng S, Huang L, Luo S, Wu X, et al. Flotillin-1 enhances radioresistance through reducing radiation-induced DNA damage and promoting immune escape via STING signaling pathway in non-small cell lung cancer. Cancer Biol Ther. 2023 Dec 31;24(1):2203332.

80. Ludwig A, Otto GP, Riento K, Hams E, Fallon PG, Nichols BJ. Flotillin microdomains interact with the cortical cytoskeleton to control uropod formation and neutrophil recruitment. J Cell Biol. 2010 Nov 15;191(4):771–81.

81. Funicello M, Novelli M, Ragni M, Vottari T, Cocuzza C, Soriano-Lopez J, et al. Cathepsin K null mice show reduced adiposity during the rapid accumulation of fat stores. PloS One. 2007 Aug 1;2(8):e683.

82. Jiang H, Cheng XW, Shi GP, Hu L, Inoue A, Yamamura Y, et al. Cathepsin K-mediated Notch1 activation contributes to neovascularization in response to hypoxia. Nat Commun. 2014 Jun 4;5:3838.

83. Desmazes C, Galineau L, Gauthier F, Brömme D, Lalmanach G. Kininogen-derived peptides for investigating the putative vasoactive properties of human cathepsins K and L. Eur J Biochem. 2003 Jan;270(1):171–8.

84. Hao L, Chen J, Zhu Z, Reddy MS, Mountz JD, Chen W, et al. Odanacatib, A Cathepsin K-Specific Inhibitor, Inhibits Inflammation and Bone Loss Caused by Periodontal Diseases. J Periodontol. 2015 Aug;86(8):972–83.

85. Hao L, Zhu G, Lu Y, Wang M, Jules J, Zhou X, et al. Deficiency of cathepsin K prevents inflammation and bone erosion in rheumatoid arthritis and periodontitis and reveals its shared osteoimmune role. FEBS Lett. 2015 May 22;589(12):1331–9.

86. Wu N, Wang Y, Wang K, Zhong B, Liao Y, Liang J, et al. Cathepsin K regulates the tumor growth and metastasis by IL-17/CTSK/EMT axis and mediates M2 macrophage polarization in castration-resistant prostate cancer. Cell Death Dis. 2022 Sep 22;13(9):813.

87. Ding Y, Li Z, Wang H, Wang Q, Jiang H, Yu Z, et al. CTSK and PLAU as Prognostic Biomarker and Related to Immune Infiltration in Pancreatic Cancer: Evidence from Bioinformatics Analysis and qPCR. Int J Genomics. 2023;2023:3914687.

88. Shen Y, Dong Z, Fan F, Li K, Zhu S, Dai R, et al. Targeting cytokine-like protein FAM3D lowers blood pressure in hypertension. Cell Rep Med. 2023 Jun 20;4(6):101072.

89. Chen L, Lin J, Lan B, Xiong J, Wen Y, Chen Y, et al. FAM3D as a Prognostic Indicator of Head and Neck Squamous Cell Carcinoma Is Associated with Immune Infiltration. Comput Math Methods Med. 2022;2022:5851755.

90. Peng X, Xu E, Liang W, Pei X, Chen D, Zheng D, et al. Identification of FAM3D as a new endogenous chemotaxis agonist for the formyl peptide receptors. J Cell Sci. 2016 May 1;129(9):1831–42.

91. Liang W, Peng X, Li Q, Wang P, Lv P, Song Q, et al. FAM3D is essential for colon homeostasis and host defense against inflammation associated carcinogenesis. Nat Commun. 2020 Nov 20;11(1):5912.

92. Wadwa J, Chu YH, Nguyen N, Henson T, Figueroa A, Llanos R, et al. Effects of ATP7A overexpression in mice on copper transport and metabolism in lactation and gestation. Physiol Rep. 2014 Jan 1;2(1):e00195.

93. Wang Y, Zhu S, Hodgkinson V, Prohaska JR, Weisman GA, Gitlin JD, et al. Maternofetal and neonatal copper requirements revealed by enterocyte-specific deletion of the Menkes disease protein. Am J Physiol Gastrointest Liver Physiol. 2012 Dec 1;303(11):G1236-1244.

94. Xie L, Collins JF. Transcriptional regulation of the Menkes copper ATPase (Atp7a) gene by hypoxia-inducible factor (HIF2{alpha}) in intestinal epithelial cells. Am J Physiol Cell Physiol. 2011 Jun;300(6):C1298-1305.

95. Ash D, Sudhahar V, Youn SW, Okur MN, Das A, O’Bryan JP, et al. The P-type ATPase transporter ATP7A promotes angiogenesis by limiting autophagic degradation of VEGFR2. Nat Commun. 2021 May 25;12(1):3091.

96. Ozumi K, Sudhahar V, Kim HW, Chen GF, Kohno T, Finney L, et al. Role of copper transport protein antioxidant 1 in angiotensin II-induced hypertension: a key regulator of extracellular superoxide dismutase. Hypertens Dallas Tex 1979. 2012 Aug;60(2):476–86.

97. Sudhahar V, Das A, Horimatsu T, Ash D, Leanhart S, Antipova O, et al. Copper Transporter ATP7A (Copper-Transporting P-Type ATPase/Menkes ATPase) Limits Vascular Inflammation and Aortic Aneurysm Development: Role of MicroRNA-125b. Arterioscler Thromb Vasc Biol. 2019 Nov;39(11):2320–37.

98. White C, Lee J, Kambe T, Fritsche K, Petris MJ. A Role for the ATP7A Copper-transporting ATPase in Macrophage Bactericidal Activity*. J Biol Chem. 2009 Dec 4;284(49):33949–56.

99. Li W, Zhu Z, Cao W, Yang F, Zhang X, Li D, et al. Esterase D enhances type I interferon signal transduction to suppress foot-and-mouth disease virus replication. Mol Immunol. 2016 Jul;75:112–21.

100. Habryka A, Gogler-Pigłowska A, Sojka D, Kryj M, Krawczyk Z, Scieglinska D. Cell type-dependent modulation of the gene encoding heat shock protein HSPA2 by hypoxia-inducible factor HIF-1: Down-regulation in keratinocytes and up-regulation in HeLa cells. Biochim Biophys Acta. 2015 Sep;1849(9):1155–69.

101. Xia LM, Tian DA, Zhang Q, Yan W, Zhu Q, Luo M, et al. [Hypoxia induces heat shock protein HSP70-2 expression in a HIF-1 dependent manner]. Zhonghua Gan Zang Bing Za Zhi Zhonghua Ganzangbing Zazhi Chin J Hepatol. 2009 Mar;17(3):207–12.

102. Albrecht I, Bieri R, Leu A, Granacher P, Hagmann J, Kilimann MW, et al. Paralemmin-1 is expressed in lymphatic endothelial cells and modulates cell migration, cell maturation and tumor lymphangiogenesis. Angiogenesis. 2013 Oct;16(4):795–807.

103. Li J, Luo X, Xiao X, Zhang X, Qi H, Liu X, et al. Decreased expression of Wiskott-Aldrich syndrome protein family verprolin-homologous protein 2 may be involved in the development of pre-eclampsia. Reprod Biomed Online. 2014 Jan;28(1):70–9.

104. Yamazaki D, Suetsugu S, Miki H, Kataoka Y, Nishikawa SI, Fujiwara T, et al. WAVE2 is required for directed cell migration and cardiovascular development. Nature. 2003 Jul 24;424(6947):452–6.

105. Arkorful MA, Noren Hooten N, Zhang Y, Hewitt AN, Barrientos Sanchez L, Evans MK, et al. MicroRNA-1253 Regulation of WASF2 (WAVE2) and its Relevance to Racial Health Disparities. Genes. 2020 May 20;11(5).

106. Serneels L, Dejaegere T, Craessaerts K, Horré K, Jorissen E, Tousseyn T, et al. Differential contribution of the three Aph1 genes to gamma-secretase activity in vivo. Proc Natl Acad Sci U S A. 2005 Feb 1;102(5):1719–24.

107. Heitzig N, Brinkmann BF, Koerdt SN, Rosso G, Shahin V, Rescher U. Annexin A8 promotes VEGF-A driven endothelial cell sprouting. Cell Adhes Migr. 2017 May 4;11(3):275–87.

108. Lash GE, Otun HA, Innes BA, Bulmer JN, Searle RF, Robson SC. Inhibition of trophoblast cell invasion by TGFB1, 2, and 3 is associated with a decrease in active proteases. Biol Reprod. 2005 Aug;73(2):374–81.

109. Brkić J, Dunk C, O’Brien J, Fu G, Nadeem L, Wang YL, et al. MicroRNA-218-5p Promotes Endovascular Trophoblast Differentiation and Spiral Artery Remodeling. Mol Ther J Am Soc Gene Ther. 2018 Sep 5;26(9):2189–205.

110. Gong Y, Li X, Xie L. Circ_0001897 regulates high glucose-induced angiogenesis and inflammation in retinal microvascular endothelial cells through miR-29c-3p/transforming growth factor beta 2 axis. Bioengineered. 2022 May;13(5):11694–705.

111. Zhang H, Akman HO, Smith ELP, Zhao J, Murphy-Ullrich JE, Batuman OA. Cellular response to hypoxia involves signaling via Smad proteins. Blood. 2003 Mar 15;101(6):2253–60.

112. Ledbetter S, Kurtzberg L, Doyle S, Pratt BM. Renal fibrosis in mice treated with human recombinant transforming growth factor-beta2. Kidney Int. 2000 Dec;58(6):2367–76.

113. Sanjabi S, Oh SA, Li MO. Regulation of the Immune Response by TGF-β: From Conception to Autoimmunity and Infection. Cold Spring Harb Perspect Biol. 2017 Jun 1;9(6).

114. Tu Y, Han J, Dong Q, Chai R, Li N, Lu Q, et al. TGF-β2 is a Prognostic Biomarker Correlated with Immune Cell Infiltration in Colorectal Cancer: A STROBE-compliant article. Medicine (Baltimore). 2020 Nov 13;99(46):e23024.

115. Yang X, Zheng E, Ma Y, Chatterjee V, Villalba N, Breslin JW, et al. DHHC21 deficiency attenuates renal dysfunction during septic injury. Sci Rep. 2021 May 27;11(1):11146.

116. Marin EP, Jozsef L, Di Lorenzo A, Held KF, Luciano AK, Melendez J, et al. The Protein Acyl Transferase ZDHHC21 Modulates α1 Adrenergic Receptor Function and Regulates Hemodynamics. Arterioscler Thromb Vasc Biol. 2016 Feb;36(2):370–9.

117. Brunetti-Pierri N, Mian A, Luetchke R, Graham BH. Intrauterine growth retardation and placental vacuolization as presenting features in a case of GM1 gangliosidosis. J Inherit Metab Dis. 2007 Oct;30(5):823.

118. Szychowski KA, Gmiński J. The VGVAPG Peptide Regulates the Production of Nitric Oxide Synthases and Reactive Oxygen Species in Mouse Astrocyte Cells In Vitro. Neurochem Res. 2019 May;44(5):1127–37.

119. Chen C, Wang X, Li Y, Zhao T, Wang H, Gao Y, et al. Hypobaric hypoxia causes low fecundity in zebrafish parents and impairment of skeletal development in zebrafish embryos and rat offspring. Reprod Toxicol Elmsford N. 2024 Aug;127:108603.

120. Zhao K, Erb U, Hackert T, Zöller M, Yue S. Distorted leukocyte migration, angiogenesis, wound repair and metastasis in Tspan8 and Tspan8/CD151 double knockout mice indicate complementary activities of Tspan8 and CD51. Biochim Biophys Acta Mol Cell Res. 2018 Feb;1865(2):379–91.

121. Porter L, Minaisah RM, Ahmed S, Ali S, Norton R, Zhang Q, et al. SUN1/2 Are Essential for RhoA/ROCK-Regulated Actomyosin Activity in Isolated Vascular Smooth Muscle Cells. Cells. 2020 Jan 6;9(1).

122. Chen X, Liu M. CircATRNL1 increases acid-sensing ion channel 1 to advance epithelial-mesenchymal transition in endometriosis by binding to microRNA-103a-3p. Reprod Biol. 2022 Jun;22(2):100643.

123. Wang D, Luo Y, Wang G, Yang Q. CircATRNL1 promotes epithelial-mesenchymal transition in endometriosis by upregulating Yes-associated protein 1 in vitro. Cell Death Dis. 2020 Jul 29;11(7):594.

124. Shaw IW, Kirkwood PM, Rebourcet D, Cousins FL, Ainslie RJ, Livingstone DEW, et al. A role for steroid 5 alpha-reductase 1 in vascular remodeling during endometrial decidualization. Front Endocrinol. 2022;13:1027164.

125. Chakraborty S, Ain R. NOSTRIN: A novel modulator of trophoblast giant cell differentiation. Stem Cell Res. 2018 Aug;31:135–46.

126. Madeja Z, Yadi H, Apps R, Boulenouar S, Roper SJ, Gardner L, et al. Paternal MHC expression on mouse trophoblast affects uterine vascularization and fetal growth. Proc Natl Acad Sci U S A. 2011 Mar 8;108(10):4012–7.

127. Zhang C, Zhao D. MicroRNA-362-5p promotes the proliferation and inhibits apoptosis of trophoblast cells via targeting glutathione-disulfide reductase. Bioengineered. 2021 Dec;12(1):2410–9.

128. Biró O, Nagy B, Rigó JJ. Identifying miRNA regulatory mechanisms in preeclampsia by systems biology approaches. Hypertens Pregnancy. 2017 Feb;36(1):90–9.

129. Guo J, Tian T, Lu D, Xia G, Wang H, Dong M. Alterations of maternal serum and placental follistatin-like 3 and myostatin in pre-eclampsia. J Obstet Gynaecol Res. 2012 Jul;38(7):988–96.

130. Xiao H, Fu J, Liu R, Yan L, Zhou Z, Yuan J. Gastric cancer cell-derived exosomal miR-541-5p induces M2 macrophage polarization through DUSP3/JAK2/STAT3 pathway. BMC Cancer. 2024 Aug 6;24(1):957.

131. Niu ZR, Han T, Sun XL, Luan LX, Gou WL, Zhu XM. MicroRNA-30a-3p is overexpressed in the placentas of patients with preeclampsia and affects trophoblast invasion and apoptosis by its effects on IGF-1. Am J Obstet Gynecol. 2018 Feb;218(2):249.e1-249.e12.

132. Li S, Li N, Li B, Zhu L, Xu T, Wang L, et al. CircHIPK3 promotes proliferation and metastasis of villous trophoblasts through miR-30a-3p/Wnt2 axis. J Genet. 2022;101:55.

133. Mathew LK, Lee SS, Skuli N, Rao S, Keith B, Nathanson KL, et al. Restricted expression of miR-30c-2-3p and miR-30a-3p in clear cell renal cell carcinomas enhances HIF2α activity. Cancer Discov. 2014 Jan;4(1):53–60.

134. Gu F, Lu D, Zhang L. MicroRNA-30a contributes to pre-eclampsia through regulating the proliferation, apoptosis, and angiogenesis modulation potential of mesenchymal stem cells by targeting AVEN. Bioengineered. 2022 Apr;13(4):8724–34.

135. Gillet V, Ouellet A, Stepanov Y, Rodosthenous RS, Croft EK, Brennan K, et al. miRNA Profiles in Extracellular Vesicles From Serum Early in Pregnancies Complicated by Gestational Diabetes Mellitus. J Clin Endocrinol Metab. 2019 Nov 1;104(11):5157–69.

136. Ali Z, Zafar U, Tauseef A, Zaki S, Khaliq S. Micro Rna 182-3-P, 519-D-5p, 378-3p As Non-Invasive Predictors Of Preeclampsia. J Ayub Med Coll Abbottabad JAMC. 2023 Sep;35(3):437–41.

137. Abbaskhani H, Seifati SM, Salmani T, Vojdani S, Al-Rubaye S, Yaseen R, et al. Evaluating changes in the expression of BCL-2 gene, lncRNA SRA, and miR-361-3p in unexplained recurrent pregnancy loss. Nucleosides Nucleotides Nucleic Acids. 2022;41(9):891–9.

138. Soni UK, Chadchan SB, Gupta RK, Kumar V, Kumar Jha R. miRNA-149 targets PARP-2 in endometrial epithelial and stromal cells to regulate the trophoblast attachment process. Mol Hum Reprod. 2021 May 29;27(6):gaab039.

139. Xiaobo 赵肖波 Z, Qizhi H, Zhiping W, Tao D. Down-regulated miR-149-5p contributes to preeclampsia via modulating endoglin expression. Pregnancy Hypertens. 2019 Jan;15:201–8.

140. Liu R, Wang X, Yan Q. The regulatory network of lncRNA DLX6-AS1/miR-149-5p/ERP44 is possibly related to the progression of preeclampsia. Placenta. 2020 Apr;93:34–42.

141. Hu Z, Dong C, Dong Q. Circ_0015382 is associated with preeclampsia and regulates biological behaviors of trophoblast cells through miR-149-5p/TFPI2 axis. Placenta. 2021 May;108:73–80.

142. Wang P, Chen X, Chang Y, Wang Y, Xu X, Guo Y, et al. Inhibition of microRNA-149 protects against recurrent miscarriage through upregulating RUNX2 and activation of the PTEN/Akt signaling pathway. J Obstet Gynaecol Res. 2020 Dec;46(12):2534–46.

143. Law YY, Lee WF, Hsu CJ, Lin YY, Tsai CH, Huang CC, et al. miR-let-7c-5p and miR-149-5p inhibit proinflammatory cytokine production in osteoarthritis and rheumatoid arthritis synovial fibroblasts. Aging. 2021 Jul 1;13(13):17227–36.

144. Li W, Chang N, Tian L, Yang J, Ji X, Xie J, et al. miR-27b-3p, miR-181a-1-3p, and miR-326-5p are involved in the inhibition of macrophage activation in chronic liver injury. J Mol Med Berl Ger. 2017 Oct;95(10):1091–105.

145. Xie W, Li M, Xu N, Lv Q, Huang N, He J, et al. MiR-181a regulates inflammation responses in monocytes and macrophages. PloS One. 2013;8(3):e58639.

146. Su Y, Yuan J, Zhang F, Lei Q, Zhang T, Li K, et al. MicroRNA-181a-5p and microRNA-181a-3p cooperatively restrict vascular inflammation and atherosclerosis. Cell Death Dis. 2019 May 7;10(5):365.

147. Wang X, Yao F, Yang L, Han D, Zeng Y, Huang Z, et al. Macrophage extracellular vesicle-packaged miR-23a-3p impairs maintenance and angiogenic capacity of human endothelial progenitor cells in neonatal hyperoxia-induced lung injury. Stem Cell Res Ther. 2024 Sep 11;15(1):295.

148. Zhou X, Wen M, Zhang J, Long K, Lu L, Jin L, et al. Unveiling the Regulatory Role of LncRNA MYU in Hypoxia-Induced Angiogenesis via the miR-23a-3p Axis in Endothelial Cells. Cells. 2024 Jul 15;13(14).

149. Tryggestad JB, Vishwanath A, Jiang S, Mallappa A, Teague AM, Takahashi Y, et al. Influence of gestational diabetes mellitus on human umbilical vein endothelial cell miRNA. Clin Sci Lond Engl 1979. 2016 Nov 1;130(21):1955–67.

150. Ceolotto G, Giannella A, Albiero M, Kuppusamy M, Radu C, Simioni P, et al. miR-30c-5p regulates macrophage-mediated inflammation and pro-atherosclerosis pathways. Cardiovasc Res. 2017 Nov 1;113(13):1627–38.

151. Stefanski AL, Martinez N, Peterson LK, Callahan TJ, Treacy E, Luck M, et al. Murine trophoblast-derived and pregnancy-associated exosome-enriched extracellular vesicle microRNAs: Implications for placenta driven effects on maternal physiology. PloS One. 2019;14(2):e0210675.
